# Electrospun Micro/Nanofiber-Based Electrocatalysts for Hydrogen Evolution Reaction: A Review

**DOI:** 10.3390/polym16223155

**Published:** 2024-11-13

**Authors:** Xiuhong Li, Youqi He, Kai Li, Shuailong Zhang, Xinyu Hu, Yi Li, Daode Zhang, Yong Liu

**Affiliations:** 1 School of Mechanical Engineering, Hubei University of Technology, Wuhan 430000, China; 20200005@hbut.edu.cn (X.L.); 102210149@hbut.edu.cn (K.L.); 102210118@hbut.edu.cn (S.Z.); 19991012@hbut.edu.cn (X.H.); 2 Beijing Key Laboratory of Advanced Functional Polymer Composites, College of Materials Science and Engineering, Beijing University of Chemical Technology, Beijing 100029, China; yongliu@mail.buct.edu.cn

**Keywords:** electrospinning, micro/nanofibers, hydrogen evolution reaction, electrocatalysts

## Abstract

Hydrogen is regarded as an ideal energy carrier to cope with the energy crisis and environmental problems due to its high energy density, cleanliness, and renewability. Although there are several primary methods of industrial hydrogen production, hydrogen evolution reaction (HER) is an efficient, eco-friendly, and sustainably green method for the preparation of hydrogen which has attracted considerable attention. However, this technique is characterized by slow reaction kinetics and high energy potential owing to lack of electrocatalysts with cost-effective and high performance which impedes its scale-up. To address this issue, various studies have focused on electrospun micro/nanofiber-based electrocatalysts for HER due to their excellent electron and mass transport, high specific surface area, as well as high porosity and flexibility. To further advance their development, recent progress of highly efficient HER electrospun electrocatalysts is reviewed. Initially, the characteristics of potential high-performance electrocatalysts for HER are elucidated. Subsequently, the advantages of utilizing electrospinning technology for the preparation of electrocatalysts are summarized. Then, the classification of electrospun micro/nanofiber-based electrocatalysts for HER are analyzed, including metal-based electrospun electrocatalyst (noble metals and alloys, transition metals, and alloys), metal–non-metal electrocatalysts (metal sulfide-based electrocatalysts, metal oxide-based electrocatalysts, metal phosphide-based electrocatalysts, metal nitride-based electrocatalysts, and metal carbide-based electrocatalysts), metal-free electrospun micro/nanofiber-based electrocatalysts, and hybrid electrospun micro/nanofiber-based electrocatalysts. Following this, enhancement strategies for electrospun micro/nanofiber-based electrocatalysts are discussed. Finally, current challenges and the future research directions of electrospun micro/nanofiber-based electrocatalysts for HER are concluded.

## 1. Introduction

In the process of rapid social development, energy remains one of the fundamental elements essential for human life, particularly fossil fuels, such as coal, petroleum, and natural gas. Their extensive use has not only damaged the ecological environment vital for human existence but triggered a series of problems, including energy shortages, posing severe crises to the sustainable development of human society [1]. Consequently, the development of renewable energy sources, such as solar, wind, and marine energy, is of great significance for adjusting the energy structure, protecting the ecological environment, and promoting sustainable development [2,3]. However, these renewable energy sources are inevitably affected by uncontrollable natural factors, such as climate and geography, which impede the continuity of energy supply, resulting in low energy utilization rates [4]. The scarcity of fossil fuels and the limitations of the aforementioned renewable energy sources has greatly hindered the advancement of human society. Therefore, nations worldwide and international organizations have progressively begun to promote the transformation of the energy structure to reduce dependence on fossil fuels and advance the development of clean energy and sustainable energy technologies [5]. In particular, the progress of clean energy holds great significance for substituting increasingly depleted fossil fuels, mitigating environmental pollution, and advancing sustainable development.

Compared to traditional fossil fuels, hydrogen has a variety of applications, serving not only as a fuel but as an industrial raw material [6]. The direct combustion of hydrogen can produce energy, with the only by-product being environmentally friendly water. Particularly noteworthy is its high energy density of approximately 142.3 MJ/kg. As a substance with the highest energy density among chemical fuels and zero-pollution combustion products, hydrogen is considered the most ideal clean energy carrier, garnering widespread attention from researchers [7,8,9]. Currently, the primary methods of industrial hydrogen production are mainly steam methane reforming and coal gasification. However, these techniques are energy-intensive, inefficient, and unfriendly to the environment, severely impeding their scale-up for industrial production [10,11,12,13]. Other hydrogen production means include biomass energy, solar energy, wind energy, and electrocatalytic water splitting [14,15,16]. The former are limited by the uncertainties of climate and natural resources [17,18]. However, electrocatalytic water splitting for hydrogen production has attracted considerable attention due to its high Faraday efficiency, environmental friendliness, and green sustainability [19,20]. It is one of the cleanest means of hydrogen production, where water is the primary substance involved in the reaction, given that water is abundant on Earth. The electrolysis of water produces hydrogen and hydrogen then combusts to regenerate water, which represents a truly green and sustainable cycle [21]. However, the efficiency of hydrogen production from water electrolysis is low, which results in slow reaction kinetics, leading to a small application range of water electrolysis in the entire field of hydrogen production [22,23]. Water electrolysis consists of two half-reactions: the hydrogen evolution reaction (HER) at the cathode and the oxygen evolution reaction (OER) at the anode [24,25,26]. Theoretically, the voltage required for electrocatalytic water splitting is 1.23 V. In practice, however, the voltage needed in commercial electrolysis cells exceeds 1.23 V [27]. This high overpotential significantly hinders its rapid development and practical application [28]. It is known that both reactions are highly dependent on electrocatalysts. Therefore, continuously developing and selecting suitable catalyst systems to enhance energy conversion efficiency is key to achieving large-scale hydrogen production [29,30]. Generally, the most commonly used electrocatalysts are based on precious metals, such as platinum (Pt), iridium oxide (IrO_2_), and ruthenium oxide (RuO_2_). Under acidic and neutral conditions, such precious metal electrocatalysts are required to accelerate the reaction [31,32]. Extensive research confirms that the most effective electrocatalysts for HER are Pt-based. Nevertheless, their high cost and low abundance pose limitations on their widespread application [33,34]. Thus, exploring efficient and cost-effective electrocatalysts is of great significance for the production of hydrogen via HER [10,35,36].

Currently, it is acknowledged that the chemical composition and micro/nanostructure of electrocatalysts play pivotal roles in determining the efficiency of HER [32,37,38]. So, the optimal design of electrocatalysts’ micro/nanostructure constitutes one of the effective methods for enhancing the electrocatalytic activity of HER. Although most existing electrocatalysts exhibit satisfactory electrochemical performance, there are several issues. Firstly, the scarcity and cost of precious metal-based electrocatalysts hinder their large-scale commercialization. Secondly, some two-dimensional electrocatalysts are generally prepared by the chemical etching or mechanical exfoliation of three-dimensional materials which are limited to those with weaker interlayer forces [39]. Lastly, traditional three-dimensional electrocatalysts are plagued by complex synthesis techniques and difficulties in their structural control [40,41]. By contrast, one-dimensional micro/nanomaterials, typically fibrous or tubular in structure with a wide range of diameters, are often endowed with unique size effects, surface effects, quantum size effects, and macroscopic quantum tunnelling effects due to their small size, large surface area, and high aspect ratio [42,43]. Moreover, their special characteristics can facilitate the rapid electron and mass transport along a controllable direction. As a result, they are extensively developed as efficient electrocatalysts for energy conversion and storage [44]. Among many types of micro/nano-electrocatalysts, those based on electrospinning technology possess excellent conductivity, unique morphology, and a large specific surface area. Those features can effectively simplify the electron transfer pathways, reduce internal resistance, and accelerate the diffusion of gases and permeation of electrolytes during the reaction process [45,46,47]. Meanwhile, electrospun micro/nanofiber-based electrocatalytic materials can expose more catalytically active sites, which are applicable to HER, OER, and other types of electrocatalytic reactions. It is evident that nanofiber-based electrocatalytic materials developed by electrospinning technology show a broad prospect in catalytic area.

To better promote the development of electrospun electrocatalysts for HER, this article firstly elucidates the characteristics of potential high-performance electrospun HER electrocatalysts. Subsequently, the advantages of utilizing electrospinning technology for the preparation of electrocatalysts are summarized. Then, the research progress on different kinds of electrospun HER electrocatalysts are summarized, including metal-based electrospun electrocatalyst (noble metals and alloys, transition metals and alloys), metal–non-metal electrocatalysts (metal sulfide-based electrocatalysts, metal oxide-based electro-catalysts, metal phosphide-based electrocatalysts, metal nitride-based electrocatalysts, metal car-bide-based electrocatalysts), metal-free electrospun micro/nanofiber-based electrocatalysts, and hybrid electrospun micro/nanofiber-based electrocatalysts. Following this, enhancement strategies for electrospun micro/nanofiber-based electrocatalysts are discussed. Finally, current challenges and the future research directions of electrospun micro/nanofiber-based electrocatalysts for HER are concluded, offering guidance for developing more efficient, eco-friendly electrospun electrocatalysts.

## 2. Properties for a Potential Effective Electrocatalyst for HER

**Figure 1:**
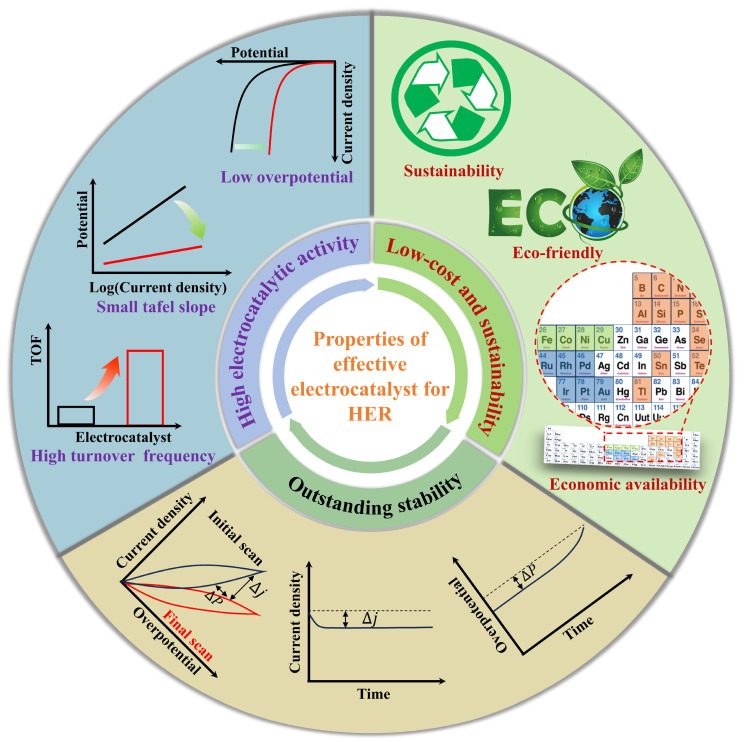
A schematic diagram of properties for a potential effective electrocatalyst for HER (The red curve in high catalytic activity indicates the curve after performance enhancement, and in stability, the red curve indicates the last cycle voltammetry curve).

Generally speaking, the high energy barrier of HER is the fundamental reason for its slow reaction kinetics. To reduce the energy barrier required for the reaction, the use of highly active electrocatalysts is the key to improving catalytic efficiency. As shown in Figure 1, an excellent potentially effective catalyst for HER should possess the following attributes: high electrocatalytic activity, outstanding stability, low cost, and sustainability [48]. These characteristics collectively determine the comprehensive performance of an electrocatalyst. Electrocatalytic activity is the core metric for judging the quality of an electrocatalyst, influencing electrocatalytic efficiency and the level of energy consumption. Stability is the other crucial feature for electrocatalysts, reflecting the ability of an electrocatalyst to maintain its activity over prolonged use. Meanwhile, cost and sustainability determine the feasibility of scaling up the electrocatalyst for industrial production. The following is a detailed discussion of these indicators.

### 2.1. High Electrocatalytic Activity

Electrocatalytic activity is typically assessed through thermodynamic and kinetic parameters. Thermodynamically, current density benchmarks of 10 mA cm^−2^ or 100 mA cm^−2^ are often employed [49]. Under these criteria, the lower the overpotential required, the less energy is consumed, and the higher the electrocatalytic activity is. Kinetically, parameters, such as the Tafel slope, exchange current density (j_0_), and turnover frequency (TOF), are used to evaluate the performance of electrocatalytic activity for an electrocatalyst. The Tafel slope is derived from the equation:

η = blogj + a

where η and j represent the overpotential and the current density. b and a are the Tafel slope and a constant, respectively. A smaller Tafel slope indicates faster charge transfer kinetics [50]. When η is equal to 0, a larger j_0_ signifies a better electrocatalytic activity [51]. TOF is used to indicate the intrinsic catalytic activity of the electrocatalyst. The higher it is, the better the electrocatalytic activity of the electrocatalyst.

### 2.2. Outstanding Stability

In addition to high electrocatalytic activity, the stability of an electrocatalyst is another critical evaluation dimension, determining whether the electrocatalyst can meet the demands of industrial production or not. There are mainly two assessment methods. The first is chronopotentiometry (I-t curve) or chronoamperometry (E-t curve), where a higher retention rate of current or potential over time after long-term stability testing indicates better stability. The second is cyclic voltammetry (CV) testing, where smaller losses in current density after more than 1000 cycles prove better stability. In essence, longer usability demonstrates better stability.

### 2.3. Low-Cost and Sustainability

The cost of an electrocatalyst decides its scalability for commercial use. Therefore, the used raw materials applied for an electrocatalyst should be abundant, easily accessible, and low-cost. On the premise of a high electrocatalytic activity, the lower the cost of an electrocatalyst, the greater the possibility of large-scale commercial production. The sustainability of an electrocatalyst means a significant practical importance in today’s societal development. To guarantee production demands, designing electrocatalysts that are nontoxic and harmless to the environment is an essential issue which needs to be considered.

The aforementioned properties of the potential electrocatalysts serve not only as evaluation criteria but as principles for designing and developing electrocatalysts. They are largely determined by the morphology, structure, composition, electrical conductivity, particle size, and specific surface area of an electrocatalyst [52,53]; these factors are closely related to the synthesis and preparation methods of the catalyst. Currently, common production methods for electrocatalysts include hydrothermal/solvothermal growth, electrodeposition, soft/hard templating, and electrospinning. However, the first three techniques have disadvantages, such as complex synthesis processes and long cycles, and a single method cannot unify all the aforementioned factors [54,55]. By contrast, electrospinning technology exhibits inherent benefits in preparing electrocatalysts for HER, including simplicity and tunability [56,57].

## 3. Advantages of Electrospun Micro/Nanofiber-Based Electrocatalysts

Electrospinning is a common method for preparing micro- or nanoscale fibers and is known for its flexibility and simplicity [58,59,60]. A typical setup usually consists of a high-voltage electrostatic generator, a metal nozzle, a syringe with a propulsion pump, and a grounded collector [58]. The fiber formation process involves stretching a charged polymer solution in a high-voltage field, where the electric field overcomes the surface tension of the charged jet, allowing the formation of solid fibers [61,62]. With technological advancements and the expansion of application areas, electrospinning devices have been continuously updated and iterated. Enhancements to electrospinning devices, like multi-nozzle electrospinning [63], nozzle less electrospinning [64], multi-jet electrospinning [65], and coaxial electrospinning [66,67], have been developed to achieve various fiber structures and improve efficiency. In addition to device configuration, electrospinning parameters significantly affect the morphology of fiber. These parameters can be categorized into three groups: the properties of the solution itself (such as polymer molecular mass, concentration, surface tension, and conductivity); process parameters (such as applied voltage, solution feed rate, and distance between the nozzle and the collector); and environmental conditions (such as temperature and humidity) [68]. The effects of these parameters on fiber morphology are summarized in Table 1.

**Figure 2:**
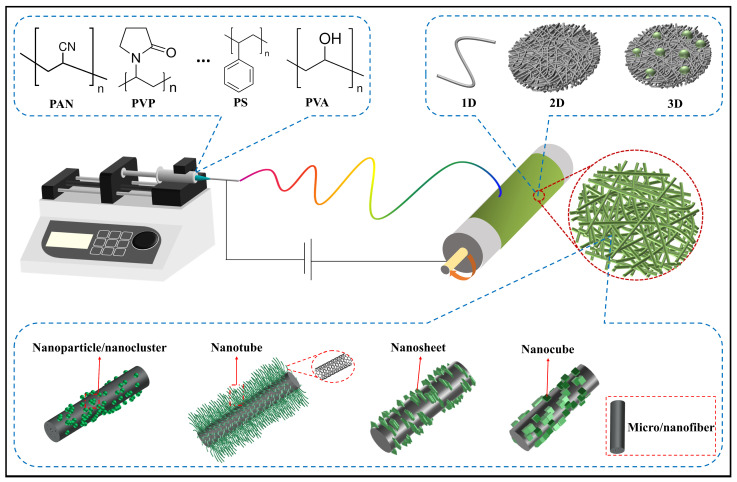
Diagram of the advantages of electrospun-based electrocatalysts.

Generally, electrospinning has clear merits to prepare electrocatalytic materials. Firstly, it is simple and controllable, allowing for the rapid production of large quantities of micro/nanofibers with tunable components; whereas traditional particle or bulk catalyst materials require complex and long preparation processes. Secondly, electrospun micro/nanofibrous materials show good conductivity after a heat treatment, and the high conductivity ensures a fast electron transfer and accelerates reaction kinetics. Notably, under the premise of selecting appropriate components, electrospinning can produce self-supporting fiber membranes with porous structure, which can facilitate the electron migration and the exposure of catalytically active sites. The structure can also compensate for the poor inherent conductivity of some active components. Lastly, electrospun micro/nanofibrous electrocatalysts possess excellent structural tunability, allowing integration of different configurations, ranging from one-dimensional (1D) to three-dimensional (3D). Through a rational structural design, rich pore channel structures and core-shell architectures can be constructed within electrospun micro/nanofibers, laying the physical foundation for enhancing the catalytic activity and efficiency of catalytic materials. Additionally, the diversities of polymer matrices available for preparing electrospun micro/nanofiber-based electrocatalytic materials offer various choices. Common polymers include polyacrylonitrile (PAN), polyvinylpyrrolidone (PVP), polystyrene (PS), polyvinyl alcohol (PVA), providing a potential for designing and preparing a large range of electrospun micro/nanofiber-based electrocatalytic materials. The advantages of electrospinning technology for preparing electrocatalysts are briefly summarized as shown in Figure 2.

## 4. Classifications of Electrospun Micro/Nanofiber-Based Electrocatalysts

**Figure 3:**
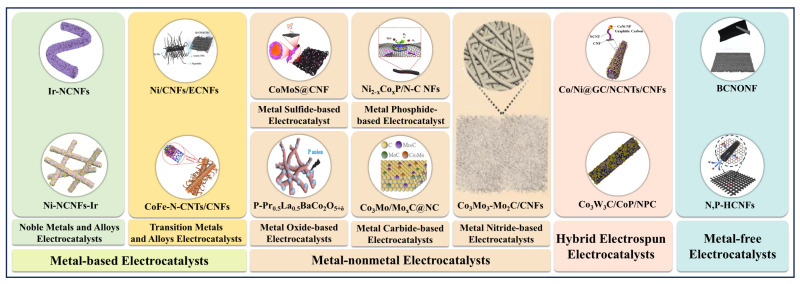
Classifications of electrospun micro/nanofiber-based electrocatalysts for HER.

In recent years, electrospinning technology has made significant progress in the preparation of electrocatalysts for electrocatalytic water splitting, especially for HER. Electrocatalysts produced by traditional synthetic methods that are large, and prone to agglomeration, lead to apparent issues with dispersibility and utilization. Electrospun micro/nanofibers used as a catalyst support not only enhance the dispersibility of catalyst but improve catalytic efficiency [83]. Moreover, by integrating additional processes, it is easy to regulate the morphology, structure, composition, and electrical conductivity of the electrospun micro/nanofiber-based electrocatalysts to modulate their catalytic activity and stability [57]. Additionally, the electrospinning process is beneficial for expanding the diversity of electrocatalysts. Normally, electrospun micro/nanofiber-based electrocatalysts for HER based on their composition can be classified into the following categories: metal-based electrospun catalysts, metal-free based electrospun electrocatalysts, and combinations of several electrospun electrocatalysts (Figure 3).

### 4.1. Metal-Based Electrospun Micro/Nanofiber-Based Electrocatalysts

For many years, there has been a dedicated effort to explore and develop electrocatalysts with high performance, low cost, and abundant reserves. Undoubtedly, noble metals are among the most active and effective electrocatalysts. Considering sustainable development, one of the current goals is to minimize the content of noble metals while developing non-noble-metal-based electrocatalysts that can rival the catalytic activity of noble metals. Carbon materials, known for their good conductivity and strong corrosion resistance, are widely used as electrocatalytic supports [84,85,86]. Among many carbon material candidates, carbon micro/nanofibers prepared via electrospinning are an ideal support for loading electrocatalysts. The carbon matrix can effectively inhibit the agglomeration of catalytic particles [87], and the high dispersibility helps to reduce the metal content in the catalyst while ensuring its high catalytic activity, thereby saving costs. Additionally, the high conductivity of the electrospun micro/nanofibers establishes a physical foundation for electron transfer. To obtain electrospun electrocatalysts with high performance and low cost, researchers have made significant explorations, such as dispersing noble metal nanoparticles like platinum (Pt) [88,89], ruthenium (Ru) [90], iridium (Ir) [91], and gold (Au) [92], in electrospun carbon nanofibers in monometallic form for HER. In addition, non-noble metal elements (e.g., iron (Fe) [93], cobalt (Co) [94,95,96], nickel (Ni) [97], copper (Cu) [98]) can be encapsulated in electrospun micro/nanofibers in monometallic or single-atom form to achieve higher electrocatalytic activity.

#### 4.1.1. Noble Metals and Alloys

Among various electrocatalysts, Pt with a Gibbs free energy close to zero is undeniably the best choice for HER electrocatalysts. Other platinum group metals, such as palladium (Pd) [99], Ru [100,101], Ir [102], and rhodium (Rh) [103,104], are also considered suitable elements for synthesizing HER electrocatalysts, but they share common drawbacks of low abundance and high cost. Generally, carbon-based micro/nano-materials possess high electrical conductivity, a large specific surface area, and abundant active sites which facilitate the loading of micro/nanocatalysts. Hence, to reduce the use of noble metals, researchers have proposed loading noble metals and their alloys onto carbon-based materials for HER electrocatalysts [105]. Particularly, electrospun micro/nanofibers are ideal carbon-based candidates for encapsulating and integrating catalyst micro/nanoparticles due to their high aspect ratio, low cost, and ease of preparation. Commonly, researchers often use electrospinning technology to coat noble metal alloys or noble metal-transition metal hybrids onto electrospun micro/nanofibers to enhance electrocatalytic performance.

**Figure 4:**
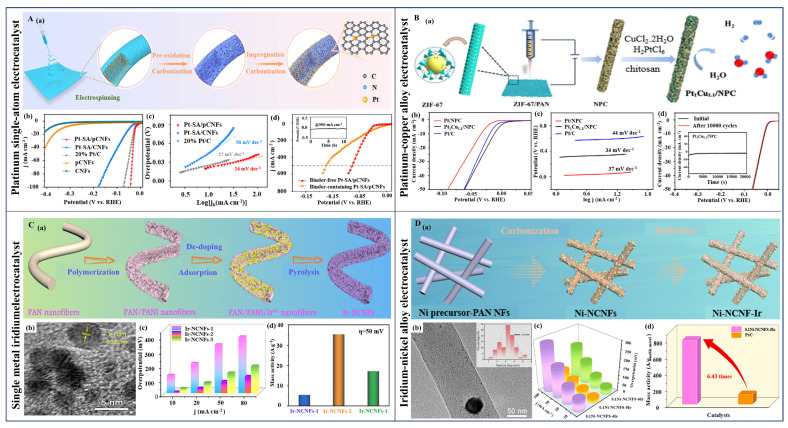
(**A**) (**a**) The fabrication process of Pt-SA/pCNFs by electrospinning. (**b**–**d**) HER electrocatalytic activities of different samples in 0.5 M H_2_SO_4_: (**b**) LSV curves, (**c**) Tafel plots and (**d**) polarization curves of the electrocatalyst for large-scale hydrogen production. (Reprinted with permission from Ref. [114], Copyright 2023, Elsevier). (**B**) (**a**) The synthetic process of Pt-Cu/NPC. (**b**–**d**) HER electrocatalytic activities of different samples in 0.5 M H_2_SO_4_: (**b**) LSV curves, (**c**) Tafel plots and (**d**) stability test (the inset is chronoamperometric response curve). (Reprinted with permission from Ref. [113], Copyright 2020, Elsevier). (**C**) (**a**) The fabrication procedure of Ir-NCNFs. (**b**) High-resolution transmisson electron microscope (HRTEM) image. (**c**,**d**) Evaluation of HER performance of Ir-NCNFs in 1.0 M KOH solution: (**c**) overpotentials at different current densities and (**d**) mass activity (MA) at the overpotential of 50 mV. (Reprinted with permission from Ref. [116], Copyright 2023, Elsevier). (**D**) (**a**) The synthetic procedure of Ni-NCNFs-Ir catalyst. (**b**) Transmisson electron microscope (TEM) image of Ir nanoparticles. (**c**,**d**) Evaluation of HER performance of Ni-NCNFs-Ir in 0.5 M H_2_SO_4_: (**c**) overpotentials at different current densities and (**d**) MA at the overpotential of 50 mV. (Reprinted with permission from Ref. [117], Copyright 2023, Elsevier).

Despite the excellent HER electrocatalytic activity of Pt and its congeners, they inherently suffer from high cost and poor stability [106]. To improve Pt’s dispersibility and prevent agglomeration during reactions, it is commonly loaded onto suitable supports. For instance, Selvan et al. prepared carbon nanofibers loaded with Pt nanoparticles on a Nb_2_O_5_ support (Nb CNF-Pt) via electrospinning [107]. The strong metal-support interaction between Nb_2_O_5_ and Pt allowed for uniform dispersion of Pt nanoparticles, favoring small particle sizes and preventing agglomeration. Even with a low Pt loading, the Nb CNF–Pt electrode exhibits a high electrocatalytic activity, requiring only 37 mV overpotential at a current density of 10 mA cm^−2^ and a Tafel slope of 38 mV dec^−1^. To further enhance the electrocatalytic activity and stability of electrospun Pt-based electrocatalysts, they also doped heteroatoms into it. Selvan et al. synthesized boron/nitrogen (B/N) co-doped carbon nanofibers loaded with Pt nanoparticles (Pt/HCNF-III) via electrospinning [108]. By optimizing the B and N doping ratio to 1:1, the Pt/HCNF-III electrocatalyst showed an HER overpotential of 54 mV and a Tafel slope of 33 mV dec^−1^, demonstrating excellent electrocatalytic performance. Alloying Pt with other transition metals (such as Co [109], Ni [45,110,111], Cu [112], etc.) is a strategy that not only improves the utilization of metal atoms but significantly reduces the amount of noble metals required. For example, Jia et al. fabricated hierarchically porous nitrogen-doped carbon nanofibers loaded with ultrafine Pt–Cu nanocrystals (Pt–Cu/NPC) using electrospinning technology (Figure 4B(a)) [113]. The prepared Pt_1_Cu_1.1_/NPC displays an excellent HER activity in an acidic environment. As shown in Figure 4B(b–d), an overpotential of only 13 mV is required to reach a current density of 10 mA cm^−2^ and a low Tafel slope of 34 mV dec^−1^ is obtained. Moreover, it demonstrates outstanding durability after 10,000 cycles of testing.

Additionally, constructing single-atom catalysts (SACs) for forming coordination structures with metal atoms can also improve utilization rates. For example, Zhang et al. prepared nitrogen-doped porous carbon nanofibers anchored with Pt single atoms (Pt-SA/pCNFs) by combining electrospinning with impregnation and thermal treatment processes (Figure 4A(a)) [114]. The firmly anchored Pt single atoms are captured by adjusting the pore of the electrospun fibers to micropores. Electrochemical tests show that the prepared Pt-SA/pCNFs exhibit excellent HER activity and stability in acidic environment, requiring only 21 mV overpotential to achieve a current density of 10 mA cm^−2^. At 100 mA cm^−2^, the overpotential is just 43 mV with a Tafel slope of 24 mV dec^−1^ (Figure 4A(b,c)). Notably, the binder-free Pt-SA/CNFs used as an independent electrode also indicates a low overpotential of just 64 mV at a large current density of 500 mA cm^−2^ (Figure 4A(d)). The strategy of employing carbon capture to anchor single atoms is equally applicable to Au. For instance, Du et al. synthesized Ru single-atom catalysts (Ru SAs/NCNF) supported on nitrogen-doped carbon nanofibers using an electrospinning method as a means for dynamic transition from clusters to single atoms [90]. The introduction of nitrogen-doping from NH_3_ by adjusting the treatment time in NH_3_ and the calcination temperature allows for the dynamic transition from Ru nanoclusters to single atoms. Electrochemical test results indicate that the optimized Ru SAs/NCNF-800-1 demonstrates an excellent HER activity in alkaline media, with an overpotential of 34 mV at a current density of 20 mA cm^−2^ and a Tafel slope of 71 mV dec^−1^. The performance of the resulting product outperforms commercial Pt/C catalysts. Additionally, chemical coupling of Ru with transition metals can reduce Ru content and enhance electrocatalytic activity. Lu et al. prepared nitrogen-doped carbon nanofibers embedded with Ru/Ni nanoparticles (RuNi-NCNFs) through electrospinning followed by a carbonization process [115]. The obtained RuNi-NCNFs exhibit efficient HER electrocatalytic activity in both acidic and alkaline conditions, with overpotentials of 35 and 23 mV, and Tafel slopes of 30 and 29 mV dec^−1^, respectively, at 10 mA cm^−2^. The synergistic effect of Ru and Ni reduced production cost while significantly enhancing electrocatalytic activity.

Iridium (Ir), another metal within the platinum group, similarly faces dispersion issues in the synthesis of high-performance electrocatalysts. Effective solutions for enhancing the dispersion of Ir-based nanoparticles have been proposed to improve their electrocatalytic performance. Yang et al. successfully anchored metallic Ir onto mesoporous carbon nanofibers (Ir-mCNFs) by introducing a certain proportion of poly(methyl methacrylate) (PMMA) as a pore-forming agent into the electrospinning precursor [91]. The Ir-mCNFs electrocatalyst with an optimized pore structure demonstrated excellent HER electrocatalytic activity in both alkaline and acidic media. At a current density of 10 mA cm^−2^, the overpotentials were as low as 28 and 39 mV, with Tafel slopes of 32 and 46 mV dec^−1^, respectively. The performance vastly exceeded that of Ir-CNFs without PMMA under identical conditions. Notably, the Ir content in Ir-mCNFs was about 6 wt%, successfully achieving the goal of low loading with a high performance. Normally, the dispersion and fixation of single-atom catalysts are achieved by designing the appropriate pore structure, while Lu et al. innovatively proposed a chelating adsorption strategy to disperse and fix Ir nanoparticles [116]. In short, ultrafine Ir nanoparticles were fixed onto polyaniline-modified N-doped carbon nanofibers (Ir-NCNFs) through electrospinning and high-temperature pyrolysis (Figure 4C(a,b)). The prepared Ir-NCNFs enhanced charge transfer and promoted the exposure of catalytically active sites, leading to accelerated reaction kinetics and exceptional electrocatalytic activity. As shown in Figure 4C(c,d), the Ir-NCNFs-2 showed a better performance than those of the other two samples. Moreover, alloying with non-noble metals can enhance dispersion and reduce usage of the noble metal. The type and ratio of non-noble metals can affect electrocatalytic activity. For instance, Lu et al. successfully loaded low-content, ultrafine Ir nanoparticles onto Ni-containing N-doped carbon nanofibers (Ni-NCNFs-Ir) using electrospinning combined with a chemical reduction deposition method (Figure 4D(a,b)) [117]. The Ni-NCNFs-Ir featured many catalytically active sites and good conductivity. The optimized Ni-NCNFs-Ir catalyst (with 6.0 wt% Ir content) exhibited outstanding HER activity in both acidic and alkaline media, with overpotentials of 22 and 25 mV at 10 mA cm^−2^, and Tafel slopes of 45.9 and 47.6 mV dec^−1^. In addition, the overpotential of 0.1Ni-NCNFs-5Ir is 83 mV at a current density of 100 mA cm^−2^, and the mass activity (MA) was much higher than that of the reference sample Pt/C at an overpotential of 50 mV (Figure 4D(c,d)). Theoretical calculations indicated that the existence of C and Ir sites, as well as electron transfer (Ni→C→N and Ir→C→N), jointly contributed to the high catalytic performance of the sample. Furthermore, the prepared 0.1Ni-NCNFs-5Ir electrocatalyst also showed long-term stability.

Palladium (Pd) possesses good catalytic activity and is more abundant, making it highly prospective for HER electrocatalysts. Although it owns relatively abundant storage, controlling its content in the electrocatalyst and achieving the optimal ratio with non-noble metals to achieve high catalytic activity are equally important. Du et al. uniformly loaded PdNi alloy nanoparticles onto carbon nanofibers (PdNi/CNF) via electrospinning and carbonization processes [118]. Due to the synergistic effect of PdNi alloy nanoparticles, the prepared PdNi/CNFs used as working electrodes displayed higher electrocatalytic activity. At a current density of 10 mA cm^−2^, the HER overpotentials for PdNi/CNFs-1:2 electrocatalysts in acidic and alkaline solutions are 55 and 187 mV, respectively, with corresponding Tafel slopes of 57 and 93 mV dec^−1^.

As a noble metal, rhodium (Rh) exhibits high electrocatalytic performance for HER. However, its strong hydrogen adsorption strength limits to some extent the efficiency of HER [119,120]. Therefore, optimizing the hydrogen adsorption strength of Rh-based electrocatalysts is an effective solution. Generally, this can be achieved by introducing elements with weaker hydrogen adsorption strength to attenuate the inherent hydrogen adsorption strength of Rh. For instance, Youngmi et al. fabricated carbon nanofibers encapsulating RhCo alloy nanotubes utilizing electrospinning and thermal reduction [121]. Electrochemical testing revealed that the amorphous RhCo alloy nanotubes possessed excellent HER activity, with an overpotential of 22 mV and a Tafel slope of 24.1 mV dec^−1^ at a current density of 10 mA cm^−2^. This was attributed to the large electrochemical surface area of the amorphous RhCo alloy and the accelerated HER reaction process at the Rh-Co interface.

With similar properties to Pt, Au shows high electrocatalytic activity. However, it tends to agglomerate, which hinders its electrocatalytic performance. To overcome this, Du et al. prepared carbon nanofibers (Au–Cu/CNFs) encapsulating Au–Cu alloy nanoparticles using electrospinning and applied them as HER electrocatalysts [122]. The study suggests that adjusting the content of Cu in the precursor could control the morphology, structure, and composition of the Au–Cu/CNFs. Additionally, due to its interwoven three-dimensional network self-supporting structure, the catalyst not only ensured electron transfer and gas desorption during the HER process but could serve as an electrode itself. Electrochemical testing showed that the hybrid exhibited excellent electrochemical activity when used as an electrode. With an Au–Cu mass ratio of 1:2, the overpotential at a current density of 10 mA cm^−2^ was 83 mV, the Tafel slope is 70 mV dec^−1^, and the exchange current density reached 0.79 mA cm^−2^.

#### 4.1.2. Transition Metals and Alloys

**Figure 5:**
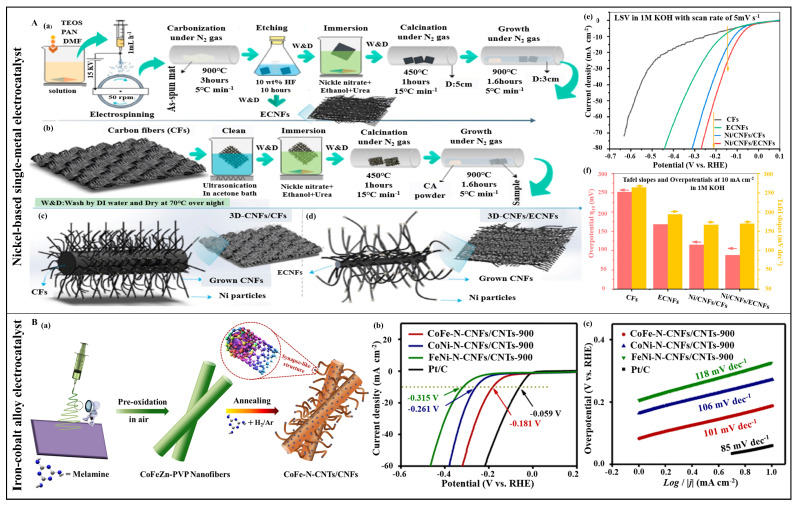
(**A**) (**a**,**b**) Comparison of preparation process of electrocatalysts: (**a**) ECNFs and 3D-Ni/CNFs/ECNFs, and (**b**) 3D-Ni/CNFs/CFs. (**c**,**d**) The diagram of final structure for samples: (**c**) 3D-Ni/CNFs/CFs and (**d**) 3D-Ni/CNFs/ECNFs. (**e**,**f**) Evaluation of HER performance of virous samples in 1 M KOH: (**e**) LSV curves, (**f**) Tafel slopes and overpotentials at a current density of 10 mA cm^−2^. (Reprinted with permission from Ref. [123], Copyright 2021, Elsevier). (**B**) (**a**) The fabrication process of CoFe-N-CNTs/CNFs. (**b**,**c**) HER electrocatalytic activities of different electrocatalysts in 1.0 M KOH: (**b**) LSV curves, (**c**) Tafel plots. (Reprinted with permission from Ref. [124], Copyright 2021, Elsevier).

Unlike single-metal catalysts formed by noble metal elements, transition metal elements are abundant and highly active. In recent years, they have been used to synthesize single metal-based electrocatalysts. For example, iron-group elements (Fe, Co, Ni, Cu, etc.) are widely used. Compared to noble metal alloy electrocatalysts, transition metal alloy electrocatalysts have significant advantages in reserves and cost. For instance, Yu et al. combined electrospinning with chemical vapor deposition (CVD) technology to prepare cactus-like three-dimensional carbon nanocomposites (Ni/CNFs/ECNFs) containing nickel (Ni) electrocatalysts on carbon fibers (CFs) and electrospun carbon nanofibers (ECNFs) [123]. The forest-like nanostructured Ni/CNFs/ECNFs demonstrated good HER performance in alkaline media, requiring only an overpotential of 88 mV to achieve a current density of 10 mA cm^−2^ (Figure 5A).

In addition to the single-metal electrocatalysts based on the aforementioned elements, researchers have also prepared alloyed metal electrocatalysts using these elements. Alloying exploits the synergistic effects of varied physicochemical properties to enhance electrocatalytic performance [125]. Typical transition metal-based alloy electrocatalysts include cobalt–nickel (Co–Ni) [126,127,128], iron–cobalt (Fe–Co) [129,130], nickel–iron (Ni–Fe) [131], nickel–molybdenum (Ni–Mo) [132], cobalt–molybdenum (Co–Mo) [133], and cobalt–copper (Co–Cu) [134] alloys. Although alloying can endow the electrocatalyst with higher activity, its performance depends on the conductivity and the exposure degree of the active sites. One solution is encapsulating alloy in carbon micro/nanofibers, ensuring high conductivity, large specific surface area, and tunable structure. For example, Zhang et al. employed electrospinning to fabricate nitrogen-doped carbon fibers with grown in situ on cobalt–nickel alloys (Co–Ni/NCNFs) [135]. The spatial confinement of carbon fibers resulted in small, dispersed alloy particles, and the Ni–Co synergy enhanced HER activity. The sample exhibited an overpotential of 61 mV and a Tafel slope of 152.3 mV dec^−1^ at 10 mA cm^−2^. The in situ growth strategy ensured excellent stability. Carbon micro/nanofibers offer internal confinement and act as substrates. Cao et al. reported a self-catalyzed graft growth strategy, where one-dimensional nitrogen-doped carbon nanotubes (N-CNTs) encapsulating CoFe alloy were directionally grown on carbon nanofibers via electrospinning technology and a pyrolysis process to form a hybrid (CoFe-N-CNTs/CNFs) (Figure 5B(a)) [124]. The prepared CoFe-N-CNTs/CNFs-900 establishes a 3D nano-forest morphology, densely distributed synapse-like structures, high porosity, and features of nitrogen doping and bimetallic active components, resulting in a high catalytic activity in an alkaline solution, as an electrode, it only needs an overpotential of 181 mV for HER to reach a current density of 10 mA cm^−2^, with a Tafel slope of 101 mV dec^−1^ (Figure 5B(b,c)). The results indicated that both spatial confinement effects and constructing three-dimensional structures can achieve a large active surface area and fully exposed active sites. Another approach is designing and constructing cluster-like electrocatalysts. Sun et al. used electrospinning to prepare an electrocatalyst by encapsulating CoMo clusters anchored on a pyrolyzed zeolitic imidazolate frameworks-8 (ZIF-8) framework in a nitrogen-doped carbon network (CoMo/CN@CNFs/MEL) [133]. The synthesized CoMo/CN@CNFs/MEL exhibited superior HER catalytic performance in an alkaline medium, requiring an overpotential of 148 mV to achieve a current density of 10 mA cm^−2^, with a Tafel slope of 98.85 mV dec^−1^. Owing to the synergistic effect of the structure and components, as well as the constrained CoMo clusters, porous nitrogen-doped carbon network, and high conductivity of carbon nanofibers, the CoMo/CN@CNFs/MEL electrocatalyst’s catalytic performance at high current densities is comparable to commercial Pt/C. Given the high porosity and dispersibility of ZIF-derived materials, incorporating them as pore-forming agents into fiber substrates is another avenue to promote the activity of transition metal electrocatalysts. Kundu et al. produced nickel–iron bimetallic zeolitic imidazolate framework-based nanofibers (NiFe-ZIF-NFs) via electrospinning [136], showing an overpotential of 290 mV at a current density of 50 mA cm^−2^ in acidic media for HER, with a Tafel slope of 89 mV dec^−1^. Furthermore, the electrocatalyst demonstrated good stability. Unlike other design protocols, pore formation via atomic radii differences has been proposed. Cheng et al. produced a porous NiMo solid-solution alloy nanofiber for HER with electrospinning and thermal treatment processes [132]. In an alkaline electrolyte, the porous nanofibers showed higher HER activity compared to Ni nanofibers, with an overpotential as low as 28 mV to achieve a current density of 10 mA cm^−2^, and a Tafel slope of 48.0 mV/dec. Notably, the overpotentials at high current densities of 100, 500, and 1000 mA cm^−2^ were 69, 208, and 300 mV, respectively. At 100 mA cm^−2^, the electrocatalyst exhibited long-term durability. In addition to these typical alloys, other types of alloys (such as ternary alloys and multi-component mixed alloys) also possess great electrocatalytic performance [137,138].

### 4.2. Metal–Non-Metal Electrospun Micro/Nanofiber-Based Electrocatalysts

Compared to compounds and alloys composed solely of precious metals or transition metals, electrocatalysts formed by metal atoms and non-metal atoms offer a richer variety and occupy a larger proportion for HER. These electrocatalysts are cheaper and provide a wider range of choices than the electrocatalysts. They can generally be divided into metal sulfide electrocatalysts, metal oxide electrocatalysts, metal phosphide electrocatalysts, metal nitride electrocatalysts, and metal carbide electrocatalysts.

#### 4.2.1. Metal Sulfide-Based Electrocatalysts

Metal sulfides, as a category of electrocatalysts with unique physical properties and excellent chemical performances, are widely used in the field of HER [139,140,141]. Generally speaking, they consist of two main categories based on their structure. One is two-dimensional materials with layered structures represented by MoS_2_ and WS_2_ [142,143]; the other shows three-dimensional spatial structures, such as CoS_2_ and NiS_2_ [144].

MoS_2_ is widely studied for its unique layered structure and good electrocatalytic activity [145,146]. These characteristics provide great flexibility, allowing optimization of its electrocatalytic activity when combined with carbon micro/nanofibers. A common method is to control the exposure of catalytic active sites through structural design. You et al. synthesized amorphous flower-like molybdenum sulfide-loaded nitrogen-doped carbon nanofiber membranes (MoS_x_@NCNFs) by combining electrospinning with carbonization and hydrothermal processes [147]. NCNFs served as a conductive substrate, accelerating electron transfer and inhibiting aggregation of MoS_x_ nanoparticles. The amorphous flower-like MoS_x_ on NCNFs exposed abundant active edge sites, thereby endowing the sample with excellent HER activity and long-term stability. MoS_x_@NCNFs required only an overpotential of 137 mV to produce a current density of 10 mA cm^−2^, with a Tafel slope of 41 mV dec^−1^. The microstructure within the electrocatalyst can also impact the electrocatalytic performance. Therefore, designing the microstructure of electrocatalysts is an effective route for their performance enhancement. Zhu et al. employed a combination of electrospinning, in situ polymerization, and carbonization processes to fabricate a core-sheath structured nanohybrid (MoS_2_@NHCF) [148], where the core and nitrogen-doped hollow carbon fibers (NHCF) were the MoS_2_ fibers and the sheath, respectively. This structure provided tunable porosity and served as a self-supporting electrode, with internal voids promoting rapid electrolyte diffusion and the NHCF sheath greatly facilitating swift electron transfer. Similarly, the core-sheath structured carbon nanofiber hybrids with CoS_2_ (CNF@CoS_2_) prepared by Liu et al. exhibited significant HER electrocatalytic activity [144]. Although core-shell structures improve the electrocatalytic activity of MoS_2_ to some extent, further enhancement requires additional active substances or conductive materials. For instance, Wu et al. synthesized nitrogen-doped carbon nanofibers with phosphorus-doped MoS_2_ nanosheets (NCNFs-MoS_2_|P) by electrospinning, serving as HER electrocatalysts [149]. Under the confinement of NCNFs, the excessive growth and aggregation of MoS_2_ nanosheets were avoided, favoring the full exposure of edge active sites, and the introduction of phosphorus atoms enhanced electron transfer. Similarly, the MoS_2_/SnO_2_ heterostructures by Liu et al. demonstrated excellent HER performance [46]. SnO_2_ embedded within the electrospun micro/nanofibers formed hollow nanotubular structures coated with MoS_2_ nanosheets, offering high specific surface area and enhanced conductivity. The experimental results indicated that the MoS_2_/SnO_2_ electrocatalyst featured a low overpotential, a small Tafel slope, and a large current density. In addition to conductive dopants, optimizing components distribution in the microstructure is effective. Zhang et al. utilized electrospinning, calcination, and hydrothermal processes to fabricate a self-supporting carbon nanofiber membrane with a “sandwich” structure (CNF/Co_3_S_4_/MoS_2_) for using as HER electrocatalyst [150]. The carbon nanofiber network functioned both as a conductive substrate and as attachment points for the growth of MoS_2_. Co_3_S_4_ accelerated the electron transfer from the conductive substrate to the electrocatalyst, while MoS_2_ provided numerous catalytic active sites. Consequently, CNF/Co_3_S_4_/MoS_2_ demonstrated exceptional electrocatalytic performance, with an overpotential of only 80 mV at a current density of 10 mA cm^−2^ in an alkaline medium, with a Tafel slope of 99.2 mV dec^−1^.

Similar to MoS_2_, WS_2_ possesses semiconductor properties, with active sites primarily located at the edges rather than the basal planes [151]. However, WS_2_ tends to aggregate and exhibits poor stability in acidic environments, leading to low interlayer charge transfer efficiency [152]. Thus, it is important to address these inherent defects to enhance the electrocatalytic performance. Hybridizing WS_2_ with carbon materials with a high specific surface area (such as CNFs) is an effective approach to solve this issue. Liu et al. applied a hydrothermal method to grow WS_2_ nanosheets in situ on flexible electrospun polyvinylidene fluoride (PVDF) fibrous membranes (e-PM@WS_2_), obtaining a stable flexible self-supporting HER electrocatalyst [153]. Electrochemical testing revealed that e-PM@WS_2_ required an overpotential of 211 mV to reach a current density of 10 mA cm^−2^ and had a Tafel slope of 101 mV dec^−1^. Introducing heteroatoms into WS_2_ is a universal method to enhance its electrocatalytic activity, yet an anion modification method is not extensively reported. Transition metal disulfide nitrides are a class of materials with high electrical conductivity, which can be metallic or semi-metallic [154,155]. Thus, employing nitrogen anion modification to boost conductivity and accelerate electron transfer is promising. For example, Du et al. synthesized nitrogen anion-modified CoWS nanosheets (N–Co_x_W_1-x_S_2_) on carbon nanofibers (CNFs) using electrospinning and thermal treatment [156]. The N–Co_x_W_1-x_S_2_ electrode required an overpotential of 93 mV at a current density of 10 mA cm^−2^, with a Tafel slope of 85 mV dec^−1^, attributed to enhanced conductivity and increased active sites. Non-layered metal sulfides such as CoS_2_ [157], NiS_2_ [140], and FeS_2_ [141] have been widely used as HER electrocatalysts, yet their electrocatalytic activity requires improvement. Mixing them with high specific surface area and high conductivity carbon materials is one of the methods to optimize performance. Zhong et al. used electrospinning and pyrolysis to embed FeS_2_ nanoparticles into porous carbon nanofibers (FeS_2_@CNFs) for use as HER electrocatalysts, which could also serve directly as self-supporting electrodes [158]. FeS_2_@CNFs showed an overpotential of 168 mV at a current density of 10 mA cm^−2^, with a Tafel slope of only 83.4 mV dec^−1^, due to high porosity facilitating electrolyte permeation and conductive carbon fibers doped with N and S preventing aggregation and structural collapse of FeS_2_ nanoparticles, enhancing mass and electron transport efficiency. After a stability test lasting 162 h, the electrode structure remained intact, indicating good stability.

**Figure 6:**
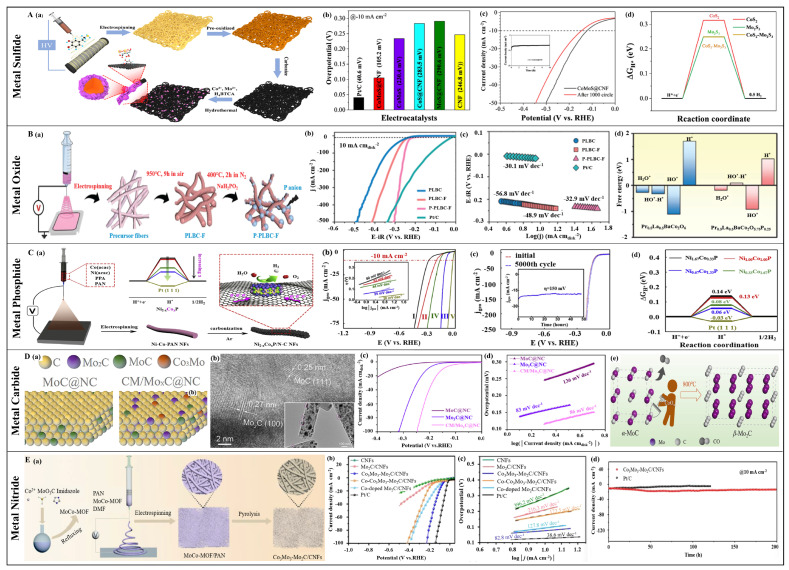
(**A**) (**a**) The synthesis process for the CoMoS@CNF catalyst. (**b**,**c**) HER performance of different electrocatalysts in 1.0 M KOH: (**b**) overpotentials at 10 mA cm^−2^ and (**c**) stability, inset: chronoamperometric response curve. (**d**) HER free energy diagrams on virous samples. (Reprinted with permission from Ref. [167], Copyright 2022, Elsevier). (**B**) (**a**) The preparation process of the P-Pr_0.5_La_0.5_BaCo_2_O_5+δ_ (P-PLBC-F) electrocatalyst. (**b**,**c**) Evaluation of HER performance of different samples in 1.0 M KOH solution: (**b**) LSV curves and (**c**) Tafel plots. (**d**) HER free energies of adsorbed intermediates (H_2_O*, HO*-H*, HO*, and H*) on two kinds of samples. (Reprinted with permission from Ref. [201], Copyright 2022, Wiley-VCH). (**C**) (**a**) Fabrication process of Ni_2-x_Co_x_P/N-C NFs. (**b**,**c**) HER performance of different samples in 0.5 M H_2_SO_4_: (**b**) HER polarization curves (the inset is virous Tafel plots) and (**c**) stability test. (**d**) HER free energy diagram on the surface of virous samples. (Reprinted with permission from Ref. [231], Copyright 2019, Elsevier). (**D**) (**a**) Surface configuration diagram of MoC@NC and CM/Mo_x_C@NC. (**b**) TEM image of CM/Mo_x_C@NC. (**c**,**d**) HER performance of different samples in 0.5 M H_2_SO_4_: (**b**) polarization curves, and (**c**) Tafel plots. (**d**) The formation of CM/Mo_x_C@NC. (Reprinted with permission from Ref. [235], Copyright 2021, Wiley). (**E**) (**a**) The preparation process of the Co_3_Mo_3_N-Mo_2_C/CNFs electrocatalyst. (**b**–**d**) Evaluation of HER performance of different samples in 0.5 M H_2_SO_4_ solution: (**b**) LSV curves and (**c**) Tafel plots, and (**d**) I-t curves. (Reprinted with permission from Ref. [236], Copyright 2023, American Chemical Society).

Compared to monometallic sulfides, the electrocatalytic activity of bimetallic sulfides depends on the functionality of each metal [159,160]. Transition metals such as Ni, Co, and Fe are used as electrocatalyst components due to their rich and tunable oxidation states and high electrocatalytic activity. For example, nickel cobalt sulfide (NiCo_2_S_4_) exhibits excellent electrical conductivity, which is comparable to that of metals [161], leading to superior electrochemical performance and stability as an electrocatalyst [162,163,164,165]. However, poor conductivity is an issue for most metal sulfides. Combining them with carbon materials to construct a conductive network is an effective solution. For example, Han et al. prepared a self-supporting carbon nanofiber network embedded with NiCo_2_S_4_ wrapped in reduced graphene oxide (rGO@NiCo_2_S_4_-CNFs) as an electrocatalyst through electrospinning, sulfidation, and surface functionalization [166]. The rGO@NiCo_2_S_4_-CNF electrode exhibited good HER electrocatalytic activity in an alkaline medium, with rGO ensuring stability, durability, and providing an effective electron transport pathway. Notably, the rGO@NiCo_2_S_4_-CNF required an overpotential of 228 mV at a high current density of 20 mA cm^−2^, with a Tafel slope of only 42.1 mV dec^−1^. Likewise, the HER electrocatalytic performance of cobalt’s monometallic sulfide also required improvement. In addition to the metal-like characteristics of cobalt nickel sulfide, it showed good electrocatalytic performance. Introducing other transition metal elements (such as Mo, W, V, etc.) into cobalt sulfide is a novel approach to enhance their electrocatalytic performance. Sun et al. prepared a self-supporting electrocatalyst by growing CoMoS on carbon nanofibers (CoMoS@CNF) using electrospinning and a hydrothermal method (Figure 6A(a)) [167]. The heterojunction between CoS_2_ and Mo_2_S_3_ significantly increased the electron mobility and reduced adsorption energy. Hence, the CoMoS@CNF catalyst required only an overpotential of 105.2 mV to reach a current density of 10 mA cm^−2^ in 1 M KOH. Even after 20 h of continuous operation at 10 mV overpotential, the electrochemical activity decayed by less than 6% (Figure 6A(b,c)). Theoretical calculations show that the heterojunction formed by CoS_2_ and Mo_2_S_3_ can effectively reduce the reaction free energy (Figure 6A(d)), explaining the high electrocatalytic activity of the CoMoS@CNF catalyst at the atomic level.

#### 4.2.2. Metal Oxide-Based Electrocatalysts

Metal oxides are a class of functional materials composed of low-cost and abundant elements [168,169,170]. However, pure metal oxides existing in bulk form are deemed unsuitable for HER due to their poor conductivity, limited electrocatalytic active sites, and poor hydrogen adsorption capacity [171,172,173,174]. By contrast, the combination of metal oxides with one-dimensional carbon fiber materials offers high thermal stability, good conductivity, and controllable morphology, which has been favored in HER processes. In recent years, metal oxide-based HER electrocatalysts have been developed, which can be broadly classified into single metal oxides [175], bimetallic oxides [176], perovskite oxides [177,178,179,180], and oxide hybrids containing oxides [181,182,183,184,185,186,187].

Transition metal, like as Fe, Co, and Ni, are known for their low-cost and high electrocatalytic activity. Their oxides constitute a class of economical and efficient HER electrocatalysts. For instance, Liu et al. used electrospinning to synthesize an amorphous, self-supporting, and flexible Fe_2_O_3_ nanofiber membrane for the first time [188], which was then employed as an HER electrocatalyst. The study found that amorphization enhanced the electrocatalytic performance of Fe_2_O_3_ and imparted good flexibility to the nanofiber membrane. Consequently, the amorphous Fe_2_O_3_ nanofiber membrane demonstrated superior electrocatalytic performance compared to its crystalline counterpart. Cobalt oxide and nickel oxide have become highly competitive candidates for the synthesis of electrocatalysts for HER. For example, Rosei and Wang fabricated a 1D/2D cobalt-based nanohybrid (CoNH) electrode by first depositing Co_3_O_4_ electrospun nanoribbons (NRs) on a carbon fiber substrate, followed by the growth of Co_3_O_4_ nanosheets via electrodeposition and ultraviolet/ozone treatment [189]. Moreover, the introduction of phosphorus source into the precursor to modify the 1D NRs further improved the electrocatalytic activity of the electrode. The electrocatalytic performance of the optimized 1D/2D CoNH electrode in a neutral medium performed as remarkably as platinum. Plus, it also exhibited long-term stability of 48 h.

Unlike other transition metal oxides, titanium dioxide (TiO_2_) has attracted attention due to its photocatalytic properties [190,191]. Its rich defect structure can enhance electrocatalytic activity when employed as a support. Zhou et al. confirmed that defect-rich TiO_2_ nanoparticles could effectively enhance the HER activity of nitrogen-rich carbon nanotube-coated cobalt nanoparticles (Co@NCT) [181]. Subsequently, He et al. proposed a lattice confinement method, which applied electrospinning to confine ultrafine Ru nanoparticles within the lattice of TiO_2_ with abundant oxygen vacancies (Ru@TiO_2_-V) [192]. The confinement effect of TiO_2_ enhanced the stability and electrocatalytic activity of Ru, yielding excellent HER performance under harsh conditions, with overpotentials of 34 and 132 mV at current densities of 10 and 100 mA cm^−2^, respectively, with a Tafel slope of 35.4 mV dec^−1^, significantly lower than 89.5 mV dec^−1^ for Ru/TiO_2_-V. Furthermore, an overpotential of only 116 mV was observed at a high current density of 800 mA cm^−2^, with long-term stability of 200 h at a current density of 300 mA cm^−2^. The exceptional performance of the synthesized electrocatalyst was mainly attributed to the lattice confinement strategy, which facilitated electron transfer from Ru to TiO_2_ and prevented the detachment and aggregation of Ru.

Bimetallic oxides are used as electrocatalysts with enhanced activity due to the synergistic effect between metals. For example, He et al. synthesized a mutually intertwined porous carbon nanofiber loaded with defect-rich Co–Ni metal oxide nanoparticles (denoted as D-CoNiO_x_-NFs) using a unique spatial confinement strategy [193]. The core of this strategy is to create lattice defects and unsaturated metal sites on the metal oxides by leveraging the differential thermal decomposition behaviors of different components within the precursor fibers. Therefore, the P-doped D-CoNiO_x_-P-NFs displayed favorable HER electrocatalytic performance. Common bimetallic oxides, such as spinel oxides, have been developed as electrocatalysts due to their good electrocatalytic activity, high abundance, and strong electrochemical stability [194,195]. Generally, they typically exhibit outstanding electrocatalytic activity for OER, though their HER performance is less satisfactory, necessitating modification of the electrocatalyst itself. For example, Subrata et al. synthesized spinel-type nickel–iron bimetallic oxide nanofibers (NiFe_2_O_4_-NFs) via electrospinning and carbonization [196]. NiFe_2_O_4_-NFs demonstrated poor HER electrocatalytic performance. To improve this, Pd nanosheets were modified on the surface of NiFe_2_O_4_-NFs to form a heterostructure, significantly enhancing performance. Experimental and theoretical calculations confirmed that the introduction of Pd into NiFe_2_O_4_-NFs was beneficial in mitigating electrocatalyst poisoning effects and enhancing its performance.

Perovskite oxides exhibit good electrocatalytic activity owing to their variable valence states, rich structural defects, and high ionic conductivity. However, their electrocatalytic activity for HER cannot compete with that of OER or oxygen reduction reaction (ORR) [197,198,199]. To enhance the HER electrocatalytic activity of perovskite oxides, strategies such as hybridization with other active substances and elemental doping have been proposed. Luo et al. developed efficient perovskite-based electrocatalysts with ORR/OER/HER trifunctionality by integrating microstructure design, bifunctional system coupling, and defect construction via electrospinning [200]. The key to their excellent HER electrocatalytic activity was the in situ coupling of mesoporous perovskite nanofibers treated with hydrogen (H_2_) (A-PBCCF-H) with Co/CoO_x_ nanoparticles. The hybrid material produced an overpotential of 224 mV at a current density of 20 mA cm^−2^ in 1.0 M KOH, with a Tafel slope of 42 mV dec^−1^. Additionally, a doping approach applied to defect-rich and structurally flexible perovskites can modulate their electronic structure and achieve superior electrochemical performance. Xia et al. synthesized P-doped Pr_0.5_La_0.5_BaCo_2_O_5+δ_ perovskite nanofibers (P-PLBC-F) using electrospinning and phosphorization processes to serve as efficient HER electrocatalysts (Figure 6B(a)) [201]. The P-PLBC-F shows a Tafel slope of 32.9 mV dec^−1^ and an overpotential of 208 mV at a current density of 10 mA cm^−2^ in an alkaline electrolyte (Figure 6B(b,c)). Notably, the overpotential is only 307 mV at a current density of 500 mA cm^−2^. The performance of P-PLBC-F was significantly improved compared to pure perovskites, which is comparable to commercial Pt/C. As illustrated in Figure 6B(d), the introduction of P reduces the Gibbs free energy and promotes the desorption of H* intermediates, thereby accelerating the reaction kinetics. In summary, high electrocatalytic activity is due to the one-dimensional nanostructure, tunable electronic structure, and optimized Gibbs free energy of H* desorption.

Hybrid materials containing oxides are highly active electrocatalysts that have been extensively explored. Youngmi et al. prepared porous iridium/iridium oxide nanofibers (Ir/IrO_2_NFs) with different Ir and IrO_2_ contents for use as HER electrocatalysts by electrospinning [202]. By adjusting calcination temperatures, the relative proportion of components in Ir/IrO_2_NFs were controlled, with higher temperatures resulting in more IrO_2_. Electrochemical tests showed that Ir/IrO_2_NFs calcined at 300 °C and exhibited the best performance in acidic media, with an overpotential of just 27 mV at 50 mA cm^−2^. The TOF of Ir/IrO_2_NF-300 was nearly 7.7 times higher than that of commercial Pt/C, with a Tafel slope as low as 30.1 mV dec^−1^ and long-term stability. Beyond applying temperature control to optimize and enhance electrocatalytic activity of this type of material, introducing another highly active substance to generate synergistic effects among multiple components is also a common and effective strategy. Yang et al. prepared a mixed electrocatalyst integrating multiple active components (Ru, RuO_2_, and MoO_3_) through electrospinning and thermal treatment (Ru–RuO_2_/MoO_3_ CNRs) [203]. Thanks to the interaction between Ru and Mo, as well as their large electrochemical surface area and high conductivity, the obtained Ru–RuO_2_/MoO_3_ CNRs-350 exhibited an excellent HER catalytic activity, whose overpotentials were as low as 9.2 and 65.4 mV at current densities of 10 and 100 mA cm^−2^, respectively. And its Tafel slope is only 37 mV dec^−1^, along with good stability in an alkaline environment.

#### 4.2.3. Metal Phosphide-Based Electrocatalysts

Metal phosphides locate near the top of the volcano plot with low hydrogen adsorption energy [204]. They are easy to prepare, and are low cost, high performance, and have strong stability. Thus, metal phosphides are considered suitable as electrocatalysts for HER [205,206]. They can be divided into transition metal phosphides (TMPs) and noble metal phosphides (NMPs).

Generally, the molecular formula for metal phosphides is represented as M_x_P_y_ (where M = Fe, Co, Ni, Cu, Mo, etc.). The M-P bonds formed in M_x_P_y_ are the fundamental reason for their high HER activity due to the ability to break chemical bonds in hydrogen molecules [207]. Cobalt phosphide has become a class of efficient HER electrocatalysts resulting from its low hydrogen adsorption free energy. However, its poor conductivity and tendency to aggregate, which leads to a reduced number of active sites, limits its overall electrochemical performance for HER. To address these issues, loading cobalt phosphide electrocatalysts onto substrates, such as carbon fibers, carbon cloth, or carbon paper, is effective to enhance their electrocatalytic performance. Electrospun carbon fibers are particularly promising due to their high conductivity, stability, and ease of processing [208,209,210,211]. Liu et al. prepared mesoporous cobalt phosphide nanotubes (CoP-NTs) with a 3D network structure through electrospinning, thermal treatment, and phosphorization [212]. To optimize the degree of phosphorization, the prepared CoP-NTs had a one-dimensional hollow tubular structure, where the hollow configuration laid the foundation for ion diffusion, electron transfer, and electrochemical interactions between the electrolyte and the electrocatalyst. Consequently, in acidic media, the CoP nanotubes exhibited an onset overpotential of only 53 mV for HER, with a Tafel slope of 50 mV dec^−1^. In addition to hollow structures, hierarchical microstructures can also enhance electrocatalytic performance. For example, Zhang et al. utilized electrospinning and pyrolysis of cobalt-based metal-organic frameworks in reductive H_2_ and the phosphidation process to embed CoP nanoparticles into carbon nanotube-grafted nanocarbon fibers (CNF@CoP-CNTs) [213]. The CNF@CoP-CNTs demonstrated excellent HER performance, with an overpotential of 65 mV and a Tafel slope of 80.79 mV dec^−1^ at a current density of 10 mA cm^−2^. Additionally, utilizing the flexibility of electrospinning to enhance the dispersion of active components is a common method to improve electrocatalytic performance. Liu et al. employed a continuous self-assembly, carbonization, and phosphatization process to modify highly dispersed Prussian blue analogue (PBA)-derived CoP nanoparticles on conductive electrospun carbon nanofibers [214]. This increased conductivity and electron transfer rate. The CoP@CNFs showed an overpotential of 85 mV at current density of 10 mA cm^−2^, with a Tafel slope of 69.9 mV dec^−1^ in an alkaline environment. In addition, the electrocatalyst particles remained stable under the protection of electrospun carbon layer.

To enhance the electrocatalytic performance of phosphide electrocatalysts, heteroatom doping is an effective method. Liu et al. prepared iron-doped cobalt phosphide nanoparticles (Fe–CoP/PCNF) on porous carbon nanofibers using electrospinning technology combined with carbonization and phosphatization processes [215]. The porous carbon nanofibers served as a conductive substrate, which not only facilitated the exposure of active sites but improved charge transfer capability. Iron doping optimized the hydrogen adsorption free energy of CoP nanoparticles, enhancing the intrinsic activity of Fe–CoP. The carbon layer’s corrosion resistance also provided good stability in long-term stability tests. In addition to heteroatom doping, coupling various electrocatalytically active components to utilize the formed heterojunction interface to regulate the electronic distribution in the vicinity, enhance electron transport efficiency, and optimize the adsorption energy of species in the reaction system can also improve electrocatalytic performance. Jiao et al. fabricated nitrogen-doped nanofibers loaded with Co and CoP nanoparticles (Co/CoP@NC) through electrospinning, localized phosphidation, and heat treatment [216]. The synergy between Co and CoP, the mass transfer through the porous N-doped conductive network, high dispersion of nanoparticles, and corrosion resistance collectively enhanced the HER activity of Co/CoP@NC. The resulting Co/CoP@NC electrocatalyst exhibited high HER electrocatalytic performance across a wide pH range, with overpotentials of 117 and 180 mV in acidic and alkaline media at a current density of 10 mA cm^−2^, respectively. The corresponding Tafel slopes were 51.3 and 60.3 mV dec^−1^, respectively.

Beyond the extensively studied cobalt-based phosphides, other transition and precious metal phosphides have been widely reported. Zhi and Hong proposed a universal method for preparing M_x_P_y_ (M = Co, Mo, Ni, and Cu) nanoparticle/carbon nanofiber composites by utilizing electrospinning technology with phytic acid as the phosphorus source [217]. The abundant and sustainable phytic acid offered an environmentally friendly approach to synthesize metal phosphides. The interwoven net-like structures facilitated electrolyte penetration, charge transport, and provided a high specific surface area. Compared to carbonized powders, the carbon nanofiber composites exhibited lower overpotentials and smaller Tafel slopes in HER, due to reduced charge transfer resistance and more active sites. Similarly, in addition to the excellent electrocatalytic activity of single-phase nickel-based phosphides, mixed-phase nickel-based phosphides have also been developed as HER electrocatalysts. Streckova et al. prepared carbon/nickel/nickel phosphide fibers using the electrospinning method [218]. The morphology, porosity, and distribution of nickel or nickel phosphide nanoparticles within the carbon fibers can be tailored by adjusting thermal treatment temperature and gas atmosphere. When used as HER electrode materials, the sample exhibited the highest HER activity in acidic media. Subsequently, the team modified carbon fiber electrode materials with copper (Cu) or copper phosphide (Cu_3_P) for HER [219]. Various thermal treatments and atmosphere reductions were explored to optimize material performance. Electrochemical testing showed that carbon fibers with Cu or Cu_3_P nanoparticles, calcined at 1000 °C under argon protection and then reduced in hydrogen, exhibited the best HER performance.

Platinum-based metals are well-known as the optimal choice for HER electrocatalysts. Ruthenium (Ru), iridium (Ir), and platinum (Pt) are all Group VIII elements and belong to the platinum group metals, sharing similar physical and chemical properties. However, there are only a few reports of Ir-based and Ru-based transition metal phosphides as HER electrocatalysts. For example, Yang et al. utilized electrospinning and thermal treatment to load Ir and its diphosphide (IrP_2_) onto 1D nanocarbon fibers (CNFs), employing them as HER electrocatalysts [220]. At a current density of 10 mA cm^−2^, the synthesized electrocatalysts demonstrated excellent electrocatalytic activity in alkaline media, with overpotentials of 22 mV for Ir-CNFs and 19 mV for IrP_2_-CNFs, and corresponding Tafel slopes of 33 mV dec^−1^ and 20 mV dec^−1^, respectively. Density functional theory (DFT) calculations indicated that the IrP_2_ (200) surface with lower water adsorption and hydrogen evolution energies displayed superior HER kinetics compared to the Ir (111) surface.

Except for monometallic phosphides, bimetallic phosphides have also captured researchers’ attention due to their unique pseudometallic behavior and improved local electronic/geometric structures, which provide superior electrocatalytic performance [221,222,223]. Recent studies have primarily focused on bimetallic phosphides composed of transition metals like Fe, Co, Ni, Mo, and W, as well as CoNiP [224,225], CoFeP [226], FeNiP [227], CoMoP [228], and MoWP [229]. Ramakrishnan et al. first adopted electrospinning to prepare carbon nanofibers loaded with NiCoP nanoparticles (NiCoP/CNF), applying these composite nanofibers as bifunctional electrocatalysts in alkaline media [230]. The widespread distribution of electrocatalytically active sites of NiCoP on the carbon fiber support, and the proximity of the metal phosphides to the apex of the volcano plot, NiCoP/CNFs demonstrated an overpotential of 130 mV at a current density of 10 mA cm^−2^ with a Tafel slope of 83 mV dec^−1^. Subsequently, some researchers proposed different means to optimize the performance of cobalt–nickel-based bimetallic phosphides. Gao et al. used a compositional optimization strategy to load bimetallic Ni_2-x_Co_x_P nanoparticles onto nitrogen-doped carbon nanofibers (Ni_2-x_Co_x_P/N-CNFs) by electrospinning and pyrolysis (Figure 6C(a)) [231]. These nanoscale particles, formed in situ within the carbon matrix, optimized electronic structure and created abundant active sites, enhancing hydrogen binding energy optimization and electrocatalytic performance across a broad pH range. Electrochemical tests showed that Ni_2-x_Co_x_P/N-CNFs reached an overpotential of 100 mV at 10 mA cm^−2^ in acidic media with a Tafel slope of 35 mV dec^−1^ (Figure 6C(b)). Excellent electrocatalytic activity was also demonstrated in alkaline and neutral media, with overpotentials of 130 and 110 mV, and corresponding Tafel slopes of 69 and 70 mV dec^−1^, respectively. Notably, Ni_2-x_Co_x_P/N-CNFs exhibited almost no difference from the initial curve after 5000 cycles and maintained stability for over 40 h at an overpotential of 150 mV (Figure 6C(c)). The optimized Ni/Co molar ratio of Ni_0.67_Co_1.33_P was close to that of Pt/C in terms of free energy, resulting in the catalyst’s superior electrocatalytic activity (Figure 6C(d)). Additionally, He et al. [232] (NiCoP@PNCNF) and Li et al. [233] (CoNiP_x_-CNF) explored the HER performance of cobalt–nickel bimetallic electrocatalysts produced by electrospinning. Beyond cobalt–nickel-based bimetallic phosphides, combinations of other transition metals to prepare bimetallic phosphides have also garnered attention. Wang and colleagues first used nitrogen-doped electrospun carbon nanofibers as a template to grow in situ CoFeP nanosheet arrays (CoFeP NS@NCNF) by a template-directed growth strategy [234]. When used as HER electrocatalysts, CoFeP NS@NCNF showed great electrocatalytic performance in alkaline conditions with an overpotential of 113 mV at 10 mA cm^−2^ and a Tafel slope of 108 mV dec^−1^. The outstanding electrochemical performance of this electrocatalyst can be attributed to its unique layered nanostructure, the high conductivity of the nanocarbon fibers, and the synergistic effect of the bimetallic phosphides.

#### 4.2.4. Metal Nitride-Based Electrocatalysts

Metal nitrides primarily refer to transition metal nitrides (TMNs), where nitrogen atoms form interstitial compounds with transition metals, which typically occupy interstitial sites in the lattice due to their smaller size [237,238,239]. The unique position of nitrogen atoms within the metal matrix endows TMNs with promising HER performance, with electronic structures similar to Pt, and they possess strong corrosion resistance, high electrical conductivity, and good stability in acidic media [240,241]. Incorporating TMNs into carbon nanofibers via electrospinning is a reliable strategy for preparing HER electrocatalysts. Mukkavilli et al. applied electrospinning to introduce metallic tantalum into polymers and synthesized Ta_3_N_5_-(O) nanofibers through ammonolysis at 800–1000 °C [242]. These nanofibers showed high electrocatalytic activity and long-term stability for HER in acidic media. However, due to the strong adsorption of hydrogen intermediates by single metal nitrides during the reaction process, the difficulty in H_2_ desorption negatively affected the electrocatalyst’s catalytic activity. Thus, constructing multi-metal nitrides to weaken the adsorption capacity for hydrogen intermediates is an effective method to enhance electrocatalytic activity. Jia et al. utilized electrospinning and pyrolysis to load ultrafine Co_3_Mo_3_N-Mo_2_C nanoparticles onto carbon nanofibers (Co_3_Mo_3_N-Mo_2_C) [236]. The synergy between Mo_2_C and Co_3_Mo_3_N optimized the electronic structure of Mo_2_C, which improved the rate of charge transfer, thereby enabling the synthesized composite to exhibit excellent HER electrocatalytic performance. Electrochemical tests revealed that the Co_3_Mo_3_N-Mo_2_C/CNF required a low overpotential of 76 mV at a current density of 10 mA cm^−2^. The Tafel slope was 82.8 mV dec^−1^. Additionally, it showed good durability with almost no decay in current density after 200 h of stability testing in acidic media.

#### 4.2.5. Metal Carbide-Based Electrocatalysts

As HER electrocatalysts, metal carbides are considered to have a broad development prospect due to their advantages such as an electronic structure similar to precious metals, good conductivity, high stability, and low cost [243,244,245]. Consequently, transition metal carbides like Mo_2_C [246,247,248], W_2_C [249], and Fe_3_C [250] have attracted extensive attention and been widely studied in recent years.

With an electronic structure similar to platinum group metals, Mo_2_C exhibits stable electrochemical performance and good corrosion resistance [251,252,253]. Hybridizing it with carbon materials can enhance the conductivity, dispersion, and the number of active sites, thereby improving electrochemical performance. Carbon fiber materials possess both high conductivity and specific surface area, making the hybridization of them with Mo_2_C an effective strategy to enhance the electrocatalytic activity [254,255,256]. Yang et al. used electrospinning and thermal treatment to prepare N and P co-doped Mo_2_C carbon nanofibers [257]. The introduction of N and P elements and the high dispersion of Mo_2_C promoted the HER process. Electrochemical tests showed that overpotentials of 196 and 107 mV in acidic and alkaline media at current density of 10 mA cm^−2^, with corresponding Tafel slopes of 81.3 and 67.5 mV dec^−1^. Moreover, the electrocatalyst displayed excellent stability. To improve the electrochemical performance of Mo_2_C, researchers introduced low content of Pt. Du et al. used a multi-molecular level confinement strategy to encapsulate ultra-low amounts of Pt atoms in α-MoC_1-x_-loaded carbon fibers (Pt/α-MoC_1-x_-CNFs) through electrospinning and thermal treatment [258]. The electrocatalyst exhibited excellent HER electrocatalytic performance in acidic conditions, with an overpotential of 38 mV and a Tafel slope of 27 mV dec^−1^ at a current density of 10 mA cm^−2^. It demonstrated a strong stability with virtually no degradation after 5000 CV cycles. The exceptional performance was due to ample exposure of active sites and the electronic modulation of α-MoC_1-x_ by Pt atoms. Beyond doping, constructing heterostructures to modulate the electronic structure can enhance activity. Lu et al. synthesized nitrogen-doped carbon nanofibers with Ni and Mo_2_C nanoparticles (Ni/Mo_2_C-NCNFs) as electrocatalysts for water electrolysis [259]. The synergy between Ni and Mo_2_C improved HER performance, with an overpotential of 143 mV and a Tafel slope of 57.8 mV dec^−1^ at a current density of 10 mA cm^−2^. This heterostructure mainly formed between metal atoms and molybdenum carbide. In addition to metal atoms, metal alloys and molybdenum carbide can also form heterostructures. Shao et al. used electrospinning to prepare a nitrogen-doped carbon nanofiber membrane loaded with cobalt–molybdenum(Co_3_Mo) coupled to Mo_x_C (CM/Mo_x_C@NC) for HER [235], as shown in Figure 6D(a,b), the atomic configurations of single-component MoC and molybdenum carbide (MoC) in coupled Co_3_Mo alloys, as well as the formation mechanism of MoC to Co_3_Mo/Mo_x_C (Figure 6D(e)). The electrocatalyst outperformed its single-component counterparts, requiring an overpotential of 114 mV and a Tafel slope of 86 mV dec^−1^ to achieve a current density of 10 mA cm^−2^ in acidic conditions (Figure 6D(c,d)). Both non-in situ and in situ characterizations revealed that the phase transition mechanism of CoO_x_ consumed the coordination carbon of α-MoC through interaction, which realized the integration of the weaker hydrogen-binding α-MoC with the stronger hydrogen-binding β-Mo_2_C, thereby enhancing the utilization rate of the active material.

### 4.3. Metal-Free Electrospun Electrocatalysts

**Figure 7:**
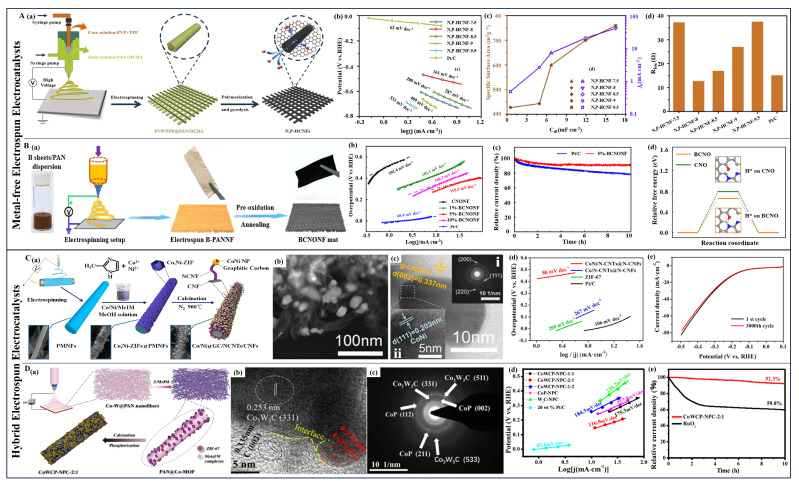
(**A**) (**a**) The fabrication procedures of N, P-HCNFs. (**b**) Tafel slopes of various electrocatalysts for HER in 0.1 M KOH solution. (**c**) BET specific surface areas, electrochemical double-layer capacitance and kinetic current density of different samples. (**d**) The ionic resistances (Rion) of various samples. (Reprinted with permission from Ref. [261], Copyright 2019, Elsevier). (**B**) (**a**) The fabrication process for the BCNONF mats. (**b**,**c**) HER electrocatalytic activities of different electrocatalysts in 1.0 M KOH: (**b**) Tafel plots and (**c**) I-t curves. (**d**) HER free energy diagrams on BCNO and CNO models. (Reprinted with permission from Ref. [263], Copyright 2021, Elsevier). (**C**) (**a**) The preparation process of Co/Ni@GC/NCNTs/CNFs based on Co,Ni-ZIFs. (**b**,**c**) HRTEM image of Co/Ni@GC/NCNTs/CNFs at (**b**) low and (**c**) high magnifications, insets: (**i**) the selected area electron diffraction (SAED) pattern and (**ii**) HRTEM image of CoNi alloy nanoparticles. (**d**,**e**) HER performance measured in 1.0 M KOH solution: (**d**) Tafel plots, and (**e**) stability. (Reprinted with permission from Ref. [264], Copyright 2022, Elsevier). (**D**) (**a**) The preparation protocol of the Co_3_W_3_C/CoP/NPC. (**b**) HRTEM image and (**c**) the SAED pattern of Co_3_W_3_C/CoP/NPC. (**d**,**e**) HER electrocatalytic activities of different samples in 1.0 M KOH: (**d**) Tafel plots, and (**e**) stability test. (Reprinted with permission from Ref. [265], Copyright 2022, Elsevier).

In recent years, a large number of transition-metal-based electrocatalysts have been developed to replace precious metal-based ones. However, their high cost hinders commercialization. Based on this consideration, researchers have explored more cost-effective metal-free electrocatalysts for HER [260]. For example, Zhi et al. first fabricated an electrocatalyst using N and P co-doped hollow carbon nanofiber membrane (N, P-HCNF) with a layered structure via coaxial electrospinning (Figure 7A(a)) [261]. The prepared N, P-HCNF possessed numerous active sites, such as defects and edges. The well-designed hollow nanostructure allowed efficient mass transfer and high specific surface area, resulting in good electrocatalytic activity. Among them, the optimized N, P-HCNF-8 demonstrates outstanding HER performance in 0.1 M KOH solution, with an overpotential of 0.55 V and a Tafel slope of 161 mV dec^−1^ at a current density of 10 mA cm^−2^ (Figure 7A(b)). These results indicate that electrocatalytic activity and mass transfer resistance in the pores are highly correlated with specific surface area (Figure 7A(c,d)). Similarly, Yang et al. synthesized nitrogen-doped carbon fibers (NCFs-800) via electrospinning and carbonization at 800 °C, serving as efficient HER electrocatalyst [262]. Thanks to nitrogen doping, the optimized NCFs-800 required overpotentials of only 114.3 mV and 198.6 mV in acidic and alkaline electrolytes, respectively, to achieve a current density of 10 mA cm^−2^, with corresponding Tafel slopes of 95.2 and 131.3 mV dec^−1^. Moreover, the HER activity maintained excellent stability after prolonged stability tests, which indicated that the carbon matrix had outstanding corrosion resistance, contributing to its exceptional durability. Heteroatom doping is a common strategy for enhancing both metal-based electrocatalysts and non-metal-based electrocatalysts. The aforementioned work highlights considerable HER performance through nitrogen/phosphorus doping or dual-atom co-doping. In contrast to single or dual-element doping, multi-atom doping exerts a more pronounced effect on modulating intrinsic activity, although such studies are less reported. Teo et al. prepared a compositionally controllable self-standing boron carbon nitride nanofiber membrane (BCNONF) via electrospinning and thermal treatment (Figure 7B(a)) [263]. The optimized BCONONF exhibited excellent electrocatalytic activity in an alkaline environment. Notably, BCONONF with 5% boron content displayed optimal electrochemical performance, requiring an overpotential of 280 mV to achieve a current density of 10 mA cm^−2^ with a Tafel slope of 115.3 mV dec^−1^ (Figure 7B(b)). Furthermore, I-t curves demonstrated a long-term stability superior to commercial Pt/C as shown in Figure 7B(c). In addition, the influence of the mechanism of the presence of B on HER performance was also investigated through theoretical calculations, and the surface free energy of BCNO was lower than that of CNO (Figure 7B(d)). The result displayed that BCNO has a stronger adsorption capacity for the intermediate H*, explaining its higher catalytic activity.

### 4.4. Hybrid Electrospun Micro/Nanofiber-Based Electrocatalysts

In recent years, the design and development of high-performance hybrid electrocatalysts have attracted increasing attention. These hybrids generally consist of two or more active components, including (Co, Co_3_O_4_, and Co_9_S_8_) CNFs [266], CoSe_2_/Co_3_S_4_@Co_3_O_4_ [267], MoS_2_@NiO [268], and Ni/Gd_2_O_3_/NiO [269]. Integrating different active components can leverage the interactions between them to modulate the electronic structure, thereby enhancing their intrinsic activity. However, components with electrocatalytic activity usually possess multiple attributes from a categorical perspective. For instance, electrocatalysts such as metal oxides, metal phosphides, metal carbides, metal sulfides, metal nitrides, and certain alloys discussed in Section 4.2 are composed of more than one active material which thus belongs to both a specific type of electrocatalyst and to multi-component electrocatalysts. Moreover, many other types of multi-component electrocatalysts have demonstrated efficient catalytic performance.

Multi-component electrocatalysts are prepared through doping. For example, Zhang et al. used electrospinning and pyrolysis to modify manganese oxide (MnO) on boron and nitrogen co-doped layered porous carbon nanofibers encapsulating iron carbide (Fe_3_C) (FeMn@BNPCFs) for the first time [270]. At an optimal heat treatment temperature of 900 °C, FeMn@BNPCFs-900 exhibited superior HER electrocatalytic performance in 1.0 M KOH, due to the three-dimensional porous structure, rapid electron transfer, abundant defects, boron and nitrogen co-doping, and well-dispersed Fe_3_C@BNC nanoparticles and MnO crystals. Metal ion doping improves conductivity and optimizes adsorption energy by altering the local electronic structure, typically resulting in better activity than non-metal atom doping. Kundu et al. prepared Ce-doped NiFe-LDH fibers (Ce@NiFe-LDH Fiber) via electrospinning [271]. Electrochemical tests showed that Ce@NiFe-LDH fibers performed a high HER activity at a current density of 10 mA cm^−2^ with an overpotential of 81 mV in an alkaline medium. While the overpotentials for Ce@NiFe-LDH powder, NiFe-LDH fibers, and NiFe-LDH powder were 120, 152, and 180 mV, respectively. The Tafel slope for Ce@NiFe-LDH fibers was 81 mV/dec. Ce doping replaced Fe ions with Ce, increasing the electrocatalyst’s surface area and active sites, thereby leading to significant electrocatalytic activity. Metals can also be co-doped with non-metal atoms to further enhance the activity of electrocatalysts. Lu et al. employed a bimetallic doping strategy to fabricate low-platinum-content Pt and Ni co-doped porous carbon nanofibers (Pt/Ni-PCNFs) via electrospinning and calcination [272]. The Pt/Ni-PCNFs with optimized platinum content exhibited an outstanding HER activity, requiring an overpotential of only 20 and 46 mV to reach a current density of 10 mA cm^−2^ in acidic and alkaline media, respectively, with corresponding Tafel slopes of 30.9 and 43.8 mV/dec. Additionally, it displayed long-term stability over 35 h. In addition to metal and non-metal ions, compounds or mixtures can also be used for doping. For example, Zhang et al. fabricated a core-shell structured electrocatalyst via electrospinning, which comprised carbon nanotubes doped with ZIF-8@ZIF-67 derived CoP/N and vertically aligned MoS_2_ nanosheets (NCT@CoP@MoS_2_) [273]. Thanks to the high porosity, hollow structure, heterojunction interfaces, and the synergy between components, the prepared NCT@CoP@MoS_2_ hybrid demonstrated a remarkable HER electrocatalytic activity in an alkaline medium, where an overpotential of 195 mV and a Tafel slope of 74 mV dec^−1^ can be achieved at a current density of 10 mA cm^−2^. Zhang et al. prepared a Co/Ni ZIFs-derived three-dimensional nanostructured electrocatalyst via electrospinning (Figure 7C(a)) [264]. As shown in the HRTEM image (Figure 7C(b)), CoNi alloy nanoparticles encapsulated in graphitic carbon layers were anchored at the tips of nitrogen-doped carbon nanotubes (Co/Ni@GC/NCNTs/CNFs) grown vertically on carbon nanofibers. The unique three-dimensional mesh structure and the introduction of carbon nanotubes facilitated mass transport as well as electron transfer. As shown in Figure 7C(c), the CoNi alloy nanoparticles were encapsulated in a carbon layer, and the protective effect of the carbon layer improved the stability of the actives, where the inset (i) SAED pattern and (ii) high-magnification HRTEM maps together verify the consistency of the CoNi alloy. Thus, the prepared Co/Ni@GC/NCNTs/CNFs exhibited superior electrocatalytic performance and excellent stability in HER (Figure 7C(d,e)). A HER overpotential of 0.15 V with a Tafel slope of 86 mV dec^−1^ was obtained at a current density of 10 mA cm^−2^ in 1.0 M KOH solution. Surface modification is another method to enhance the performance of electrocatalysts. Yang et al. fabricated Ru nanoparticle-modified NiV_2_NO heterostructured carbon nanofiber-based electrocatalysts (Ru/Ni-V_2_NO/NCNFs) via electrospinning and annealing [274]. The interaction between Ru and the Ni-V_2_NO heterostructure, the electronic structure of Ru was modulated, enhancing HER performance. On the other hand, the presence of the Ni-V_2_NO heterostructure led to a substantial increase in the electrochemically active surface area, facilitating the full exposure of active sites. Therefore, compared to electrocatalysts modified only with Ru or Ni-V_2_NO, the Ru/Ni-V_2_NO/NCNFs showed a significant improvement in HER performance, with an overpotential of just 74.5 mV and a Tafel slope of 60.7 mV dec^−1^ at a current density of 10 mA cm^−2^. Also, it demonstrated an excellent stability in an alkaline electrolyte.

Aside from previous methods, introducing suitable components and designing rational heterointerfaces can also synthesize multi-component electrocatalysts with high catalytic activity. Jiao et al. synthesized a heterostructured SrTiO_3_@MoS_2_ nanofiber composite electrocatalyst through electrospinning and hydrothermal methods [275]. Compared to SrTiO_3_ nanofibers and MoS_2_ nanosheets, the SrTiO_3_@MoS_2_ heterostructure demonstrated superior bifunctional electrocatalytic performance, requiring only an overpotential of 165 mV for HER at a current density of 10 mA cm^−2^, with a Tafel slope of 81.41 mV dec^−1^. Moreover, it showed long-term durability after 3000 cycles. Similarly, Tong et al. synthesized N and P-doped hierarchical porous carbon fibers loaded with Co_3_W_3_C/CoP nanoparticle heterostructures (Co_3_W_3_C/CoP/NPC) by using electrospinning in conjunction with pyrolysis and phosphorylation processes (Figure 7D(a)) [265]. The HRTEM image (Figure 7D(b)) and the SAED pattern (Figure 7D(c)) confirmed the existence of Co_3_W_3_C and CoP crystals and their interfaces. The Co_3_W_3_C/CoP/NPC catalysts, with abundant interfaces and active sites, exhibited good electrocatalytic activity. A HER overpotential of 139 mV and a Tafel slope of 116.9 mV dec^−1^ were achieved in alkaline electrolyte at 10 mA cm^−2^. In addition, the catalyst showed good stability, as its current density decayed by only 7.7% after 10 h of stability testing (Figure 7D(d,e)).

## 5. Enhancement Strategies for Electrospun Micro/Nanofiber-Based Electrocatalysts

**Figure 8:**
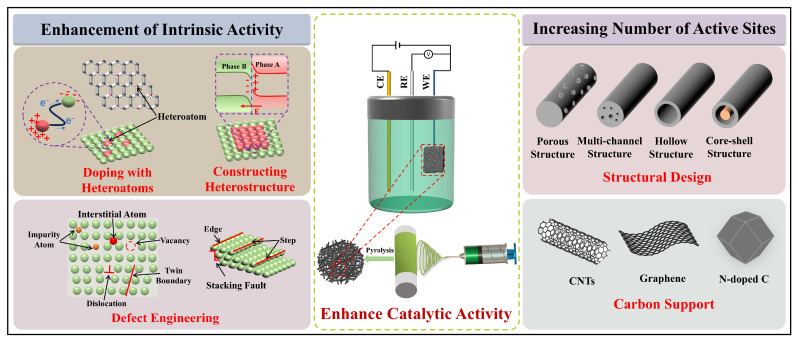
The diagram of enhancement strategies for electrocatalytic performance of electrospun micro/nanofiber-based electrocatalysts(The red arrow indicates the direction of the electric field strength and the blue arrow indicates the direction of electron migration).

Electrocatalytic water splitting for hydrogen production is a key method for obtaining green and clean energy. Designing efficient electrocatalysts is crucial to enhance hydrogen production, reduce energy consumption, lower costs, and improve efficiency. From a mechanistic standpoint, the electrocatalytic water splitting reaction fundamentally consists of the two-electron transfer of HER and the four-electron transfer of OER. The slow kinetics of these sub-reactions contribute to high overpotentials, as the efficiency of HER is dependent on the kinetics of OER. Thus, the performance of HER and OER electrocatalysts largely determines the overall performance of electrocatalytic water splitting. Optimizing and enhancing the performance of electrocatalysts is essential. Specific optimization and enhancement strategies are illustrated in Figure 8, which primarily focuses on improving the intrinsic activity of electrocatalysts and increasing the number of active electrocatalytic sites [276,277]. Different optimization strategies for various electrocatalysts can be implemented in diverse ways, as shown in Table 2.

Current strategies to enhance the intrinsic activity of electrocatalysts mainly include constructing heterostructures, doping with heteroatoms, and defect engineering. The number of active sites can also be increased by adjusting the electrocatalyst’s structure. It is noteworthy that enhancements in intrinsic activity and the increase in the number of active electrocatalytic sites of electrocatalyst are not isolated events. They can be simultaneously improved with a rational design. The strategies related to Table 2 can achieve the micro/nano structural regulation and surface performance enhancement of electrocatalysts, leading to ideal electrocatalytic performance.

### 5.1. Enhancement of Intrinsic Activity

Generally, the intrinsic activity of electrocatalysts is achieved through the regulation of electronic structure, which primarily involves constructing heterostructures, doping with heteroatoms, and defect engineering. The formation of heterointerfaces in heterostructures can generate new active centers through electronic interactions, and the interactions at these interfaces can facilitate electron transfer. Heteroatom doping can alter electron density distribution and surface activity, as well as provide additional active sites to enhance the electrocatalyst’s specific surface area. Defect engineering can adjust atomic arrangements and surface electronic structures, and even create new active sites, thereby improving the interaction of electrocatalysts with reactants. Thus, these strategies are widely used.

#### 5.1.1. Constructing Heterostructures

Heterostructured electrocatalysts have been widely investigated due to their synergistic effect arising from abundant heterointerfaces, which can promote reaction rates. The presence of heterointerfaces disrupts the original electronic equilibrium, leading to electronic reconstruction with equal positive and negative charges. For instance, Yu et al. incorporated CoSe_2_–CoO heterostructured nanoparticles within high-temperature calcined N-doped carbon fibers (CoSe_2_–CoO/NCF) using a partial selenization strategy [278]. The electronic coupling of the components in this hybrid resulted in multi-component characteristics. The heterojunctions between CoO and CoSe_2_ created numerous nanointerfaces that enhanced charge transfer and optimized reaction intermediate adsorption. Electrochemical test results indicated that CoSe_2_–CoO/NCF exhibited good bifunctional catalytic activity, characterized by low overpotential, small Tafel slope, and strong durability. Furthermore, a low voltage of just 1.604 V was required to reach a current density of 10 mA cm^−2^ when CoSe_2_–CoO/NCF was used as a full water splitting electrode.

Inducing electron reconstruction is a common method to modulate the electronic structure of electrocatalysts, achievable through various techniques. Notably, Mott–Schottky heterojunctions are extensively used to regulate electronic structures by forming metal–semiconductor interfaces. In fact, these interfaces can enhance conductivity and alter intermediate adsorption by restructuring electron cloud density, thereby improving electrocatalytic activity [279,280,281]. Tang et al. prepared bifunctional electrocatalysts by combining the Mott–Schottky effect with an electrospinning–annealing process to anchor Ni/CeO_2_ hetero-nanoparticles onto nitrogen-doped carbon nanofibers (Ni/CeO_2_@N-CNFs). Experimental results and theoretical calculations showed electrons migrated from the Ni side to the CeO_2_ side, creating holes near Ni and electron enrichment on the CeO_2_ side. This unique electron redistribution effectively enhanced the charge transfer and optimized adsorption energy, with a Gibbs free energy ΔG*H of only 0.68 eV, leading to accelerated reaction kinetics. Electrochemical tests revealed that Ni/CeO_2_@N-CNFs exhibited excellent HER and OER electrocatalytic activity in alkaline solution, with overpotentials of 100 and 230 mV at 10 mA cm^−2^ and Tafel slopes of 85.7 and 54.2 mV dec^−1^, respectively. When Ni/CeO_2_@N-CNFs were used as electrodes for electrolysis, a cell voltage of only 1.56 V was needed to achieve a current density of 10 mA cm^−2^. Plus, there was no significant degradation observed after over 55 h of testing. Additionally, heterostructure construction is not limited to metal compounds; it can involve non-metallic active substances. Han et al. constructed a three-dimensional network self-supported heterostructure electrocatalyst by coating thin layers of graphene oxide onto carbon nanofibers encapsulated with CoNi_2_S_4_ nanoparticles (CoNi_2_S_4_/CNFs) [166]. Sulfurization and surface functionalization imparted excellent electrocatalytic activity, with metallic characteristics from sulfur and graphene oxide. The conductive carbon framework and the uniformly dispersed CoNi_2_S_4_ nanoparticles protected by the outer graphene oxide layer formed a heterostructure with synergistic effect. Hence, the self-supported CoNi_2_S_4_/CNFs electrode exhibited good activity and stability for both OER and HER in an alkaline electrolyte. At a current density of 20 mA cm^−2^, the overpotentials for OER and HER were 205 and 228 mV, respectively. The corresponding Tafel slopes were 42.1 and 23.5 mV dec^−1^. When the electrodes were used for overall water splitting, a cell voltage of only 1.55 V was required to generate a current density of 10 mA cm^−2^. In summary, the types and construction methods of heterostructures are diverse, and this strategy is one of the important means to regulate electrocatalyst intrinsic activity.

#### 5.1.2. Doping with Heteroatoms

Introducing heteroatoms into electrocatalyst materials disrupts the original state of electron distribution, modulating the electronic structure and enhancing electrocatalytic activity. Doping elements can be non-metals (such as N, O, P, S, B, and Se) or metals (such as Fe, Co, Ni, and V). Generally, the electrocatalytic activity of electrocatalysts can be regulated by varying the type, concentration, and configuration of the doping elements [282]. For instance, Liu et al. synthesized an efficient HER electrocatalyst by growing phosphorus-doped NiCo_2_S_4_ nanocrystals on carbon nanofibers wrapped with carbon nanotubes (P-NiCo_2_S_4_@CNT/CNF) [163]. Carbon nanotubes enhanced the conductivity of the three-dimensional carbon framework, facilitating electron transfer within the active materials. NiCo_2_S_4_ nanoparticles were anchored onto the CNT/CNF template, exposing more catalytically active sites. Phosphorus doping improved the electronic structure of NiCo_2_S_4_, reducing the energy barrier required for the reaction. Improved conductivity, nano-architecture, and phosphorus doping optimized the electronic structure, enabling P-NiCo_2_S_4_@CNT/CNF to exhibit excellent HER performance, with an overpotential of just 74 mV at a current density of 10 mA cm^−2^. The exchange current density was 0.79 mA cm^−2^, and it showed outstanding stability.

Beyond single-element doping, multi-element doping often yields better results. For example, Mu et al. synthesized nitrogen and phosphorus-doped carbon nanofibers encapsulating Fe_3_O_4_ and FeP nanoparticles through electrospinning, pyrolysis, and phosphidation processes [283]. Notably, the synthesized electrocatalyst exhibited a good catalytic activity for ORR, HER, and OER. The microporous nanostructure within the fibers provided rapid channels for mass transfer and enhanced the corrosion resistance of the electrocatalyst, thereby improving its electrocatalytic activity and stability. Experimental results revealed that different products showed varying reactivity towards different reactions. Fe_3_O_4_ and the doped carbon with N and P contributed to the electrocatalytic behavior for ORR, OER and HER, whereas FeP was the main active substance for HER, significantly improving its performance. Thanks to the synergistic effect between the N and P-doped carbon layer and Fe_3_O_4_/FeP nanoparticles, the electrochemical performance of Fe_3_O_4_ nanoparticles was far superior to that of the N and P-doped carbon layer without Fe_3_O_4_ nanoparticles.

Similarly, multi-metal element doping is a common method to regulate electronic structures. Cheng et al. prepared Ru and Ni bimetal-doped cobalt phosphide (CoP) porous nanofibers (Ru, Ni-CoP) using electrospinning and phosphidation [284]. Compared to single-metal doping, bimetal doping significantly modulated the electronic structure of active sites, facilitating electrocatalytic water splitting by optimizing the adsorption energies of reaction intermediates. Theoretical calculations demonstrated that the hydrogen adsorption energy of Ru, Ni-CoP was close to zero, indicating excellent activity. At a current density of 10 mA cm^−2^, the HER overpotential and Tafel slope was 45 mV and 53.9 mV/dec, respectively. Similarly, employing CoP as the electrocatalytically active substance, the type and number of dopant elements distinctly affected electrocatalytic performance. Cheng et al. fabricated multi-channel hollow nanofibers doped with vanadium in CoP (MC-V-CoP) through electrospinning combined with oxidation/phosphidation [205]. The multi-channel hollow structure and V doping increased the exposure of catalytically active sites, accelerated electron transfer, and optimized the electronic structure of the active sites, thereby endowing them with exceptional HER performance. Electrochemical tests showed that MC-V-CoP hollow nanofibers achieved an overpotentials of 65 and 189 mV at high current densities of 10 mA cm^−2^ and even 300 mA cm^−2^ in an alkaline medium. This performance surpassed that of commercial 20 wt.% Pt/C catalyst under high current density (212 mV at 300 mA cm^−2^).

#### 5.1.3. Defect Engineering

Defect engineering is a common method to enhance the intrinsic activity of electrocatalysts. Defects can lead to local electron rearrangement and alter the adsorption and desorption capacities for reactants, thereby modifying the intrinsic activity and the number of active sites of electrocatalysts. Generally, there are two methods to optimize and enhance electrocatalytic activity through defect engineering. The first approach is to construct crystal defects (such as vacancies, grain boundaries, and dislocations) to improve electrocatalytic performance. For example, He et al. proposed a strategy for creating defects within an electrocatalyst by spatial confinement, where Prussian blue analogue (PBA) cubes were incorporated into PAN precursor nanofibers through electrospinning, followed by a pyrolysis and P doping (denoted as D-CoNiO_x_-P-NFs) [193]. This method utilized differential thermal behaviors during calcination: PBA expands outwardly while PAN contracts inwardly, imposing spatial constraints and inducing lattice defects and unsaturated metal sites. Consequently, the P-doped interlinked porous nanofibers encapsulating defect-rich metal oxide nanoparticles exhibited a good OER and HER performance. At a current density of 10 mA cm^−2^, the OER overpotential of D-CoNiO_x_-P-NFs was 246 mV, which was 117 mV lower than CoNiO_x_-P-NFs, with a Tafel slope of 60.5 mV dec^−1^, compared to 77.0 mV dec^−1^. Additionally, at a current density of 10 mA cm^−2^, the HER overpotential of D-CoNiO_x_-P-NFs was only 145 mV which was significantly less than the 219 mV of CoNiO_x_-P-NFs, and the Tafel slope was 76.0 mV dec^−1^, which was lower than the 87.8 mV dec^−1^ of CoNiO_x_-P-NFs. Notably, D-CoNiO_x_-P-NFs achieved a current density of 10 mA cm^−2^ at the low voltage of 1.52 V when used as a full water-splitting electrocatalyst. The other method involves creating defects at the edges of active materials to generate active sites. For instance, You et al. fabricated a hybrid composed of N-doped carbon nanofibers with ultra-thin, defects and sulfur-rich MoS_2_ nanosheets (MoS_2_/NCNFs) by electrospinning [285]. Along with N doping, the synergistic interaction between the carbon fiber substrate and MoS_2_ nanosheets endowed the material with good conductivity and stability. Also, the defect-rich and sulfur-rich MoS_2_ nanosheets possessed abundant edge active sites, conferring the synthesized electrocatalyst with a high HER activity. MoS_2_/NCNFs exhibited an overpotential of 135 mV at a current density of 10 mA cm^−2^. Furthermore, it demonstrated a low Tafel slope of 48 mV dec^−1^ and an excellent cyclic stability.

**Table 2:** Summary of reported electrochemical performances of electrospun micro/nanofiber-based electrocatalysts for HER.

Key Strategy	Electrocatalyst	η_10_ (mV)	Tafel Slope (mV/dec)	Electrolyte	Ref.
Constructing Heterostructures	CoS_2_-C@MoS_2_	173	61	0.5 M H_2_SO_4_	[286]
	rGO@CoNi_2_S_4_/CNF	228 (η_20_)	42.1	1.0 M KOH	[166]
	Co_9_S_8_/HWS_2_/CNFs	83	56	0.5 M H_2_SO_4_	[287]
		87	72	1.0 M KOH	
	CoMoS@CNF	105.2	152.8	1.0 M KOH	[167]
	Ru-Ru_2_P@CNFs	11	24.5	0.5 M H_2_SO_4_	[206]
		14	24.2	1.0 M KOH	
	CoP/Co_2_P@ACNF	133	53.5	0.5 M H_2_SO_4_	[288]
		155	88.6	1.0 M KOH	
	CoP-PrBa_0.5_Sr_0.5_Co_1.5_Fe_0.5_O_5+δ_	240	93.8	1.0 M KOH	[289]
	D-TiO_2_/Co@NCT	57.5	73.5	0.5 M H_2_SO_4_	[181]
	NiFe_2_O_4_-NFs	61	40	1.0 M KOH	[196]
	RuO_2_/Ru-CNFs	21	25.1	1.0 M KOH	[186]
	Ni/CeO_2_@N-CNFs	100	85.7	1.0 M KOH	[290]
	S-Co-NiO@N-CNF	169	86.44	1.0 M KOH	[185]
	Fe_3_C-Mo_2_C/NC	116	43	0.5 M H_2_SO_4_	[291]
	Ni/Mo_2_C-NCNFs	143	57.8	1.0 M KOH	[259]
	Sn/Mo_2_C-CNFs	144	49.8	1.0 M KOH	[255]
	Mo_2_C-CoO@N-CNFs	115	76	1.0 M KOH	[292]
	CoSe_2_–CoO/NCF	72	68.3	0.5 M H_2_SO_4_	[278]
		117	124.5	1.0 M KOH	
	SrTiO_3_@MoS_2_	165	81.41	1.0 M KOH	[275]
	Co_3_W_3_C/CoP/NPC	139	116.9	1.0 M KOH	[265]
	Ru/Ni-V_2_NO/NCNFs	74.5	60.7	1.0 M KOH	[274]
	CoSe_2_/Co_3_S_4_@Co_3_O_4_	165	117.7	1.0 M KOH	[267]
Doping with Heteroatoms	P-NiCo_2_S_4_@CNT/CNF	74	65.9	0.5 M H_2_SO_4_	[163]
	NCNFs-MoS_2_|P	98	66	0.5 M H_2_SO_4_	[149]
	NCT-NiCo_2_S_4_	183	89.8	1.0 M KOH	[293]
	(NiCo)S_2_/NCNF	177	66.9	1.0 M KOH	[294]
	SFCNF/Co_1-x_S@CoN	20	54.4	1.0 M KOH	[295]
	Ru, Ni-CoP	45	53.9	1.0 M KOH	[284]
	Fe-CoP/PCNF	151	53.9	0.5 M H_2_SO_4_	[215]
	FeCNFs-NP	149	58	0.5 M H_2_SO_4_	[283]
	MC-V-CoP	65	72.5	1.0 M KOH	[205]
	P-Pr_0.5_La_0.5_BaCo_2_O_5+δ_	224 (η_500_)	32.9	1.0 M KOH	[201]
	NiCoP@PNCNF	98	58	1.0 M KOH	[232]
	CoNiP_x_-CNFs	154	73	1.0 M KOH	[233]
	VxCo_3-x_C/CNFs	87	78	1.0 M KOH	[296]
	Co, Mo_2_C-CNF	128	60	1.0 M KOH	[254]
		206	92.8	1.0 M PBS	
	Mo_2_C/Co/CoO-NHCNFs	143	74	1.0 M KOH	[297]
	Pt/α-MoC_1-x_-CNFs	38	27	0.5 M H_2_SO_4_	[258]
	Mo_x_C-NPC	125	70.1	0.5 M H_2_SO_4_	[248]
		71	114.1	1.0 M KOH	
	Co_3_Mo/Mo_x_C@NC	114	86	0.5 M H_2_SO_4_	[235]
	Co-N-P-CNFs	248	56.14	0.5 M H_2_SO_4_	[94]
	Pt/HCNF-III	54	33	0.5 M H_2_SO_4_	[108]
	Ce@NiFe-LDH	81	81	1.0 M KOH	[271]
	0.15Co-NCNFs-5Rh	18	73.8	0.5 M H_2_SO_4_	[272]
		13	27.2	1.0 M KOH	
Defect Engineering	MoS_2_/NCNFs	135	48	0.5 M H_2_SO_4_	[285]
	Co/CoO_x_@(PrBa_0.8_Ca_0.2_)_0.95_(Co_1.5_Fe_0.5_)_0.9_	224 (η_20_)	42	1.0 M KOH	[200]
	D-CoNiO_x_-P-NFs	145	76	1.0 M KOH	[193]
	Ru@TiO_2_-V	34	35.4	1.0 M KOH	[192]
	CoP-NTs	152	50	0.5 M H_2_SO_4_	[212]
	CoP/NCF	86	55	0.5 M H_2_SO_4_	[208]
	CoP@CF	190 (η_50_)	92.6	1.0 M KOH	[209]
Hierarchical Structure	MoS_2_-CNF	120	45	0.5 M H_2_SO_4_	[145]
	CNF@CoS_2_	110	66.8	0.5 M H_2_SO_4_	[144]
	CNF/Co_3_S_4_/MoS_2_	80	99.2	1.0 M KOH	[150]
	CNF@CoP-CNTs	65	80.79	0.5 M H_2_SO_4_	[213]
	CoFeP NS@NCNF	113	108	1.0 M KOH	[234]
	PrBa_0.5_Sr_0.5_Co_2_O_5+δ_@FeOOH	280	70	0.1 M KOH	[179]
Porous Structure	Fe_3_C@FeNC-CNF	86	140	1.0 M KOH	[250]
	Mo_2_C-MCNFs	114	88	1.0 M KOH	[298]

### 5.2. Increasing the Number of Active Sites

Although much of the current research on electrocatalyst performance optimization focuses on enhancing its intrinsic activity, improving apparent activity is equally important. Apparent activity is generally achieved by increasing the number of active sites, which are usually influenced by the support, microstructure, and morphology of the electrocatalyst. To expose active sites to the greatest extent, researchers often employ hierarchical structures in electrocatalyst design. Zhang et al. fabricated a hierarchical structured CNF/Co_3_S_4_/MoS_2_ by pyrolyzing electrospun nanofibers and subsequently growing MoS_2_ in situ on N-doped nanofibers coated with Co_3_S_4_ nanoparticles through a hydrothermal method [150]. The hybrid was directly used as a self-supporting electrode. The highly conductive N-doped 3D carbon nanofiber scaffold provided channels for mass transfer and served as a substrate for the in situ growth of Co_3_S_4_ and MoS_2_ nanocrystals with rich active sites. Consequently, CNF/Co_3_S_4_/MoS_2_ exhibited outstanding electrocatalytic performance in electrolysis, requiring only 80 mV to achieve a current density of 10 mA cm^−2^ in alkaline media, with a Tafel slope of 99.2 mV dec^−1^. Moreover, this performance showed a negligible degradation after prolonged stability testing. These results underscore the importance of mass transfer and exposure of active sites.

Constructing a porous structure is an effective way to enhance mass transfer efficiency and the degree of active site exposure. Qin et al. massively produced porous carbon nanofibers with abundant channels through pyrolysis of electrospun nanofibers containing a pore-forming agent [250]. By adjusting the ratio of PAN to cellulose acetate (CA), the pore structure and quantity could be tuned. Following this exploration, a binder-free electrocatalyst was prepared, consisting of Fe_3_C encapsulated in Fe/N-doped porous carbon nanofibers with rich channel structures (Fe-CACNFs). The porous channel structure and high conductivity of the carbon fibers provided a wealth of accessible active sites and rapid mass transfer channels, leading to a good electrocatalytic activity and a remarkable stability in alkaline media. Fe-CACNFs exhibited an overpotential of 86 mV at a current density of 10 mA cm^−2^, with a Tafel slope of 140 mV dec^−1^. Additionally, the overpotential at a current density of 80 mA cm^−2^ was 440 mV.

## 6. Conclusions and Perspectives

Electrospun micro/nanofiber-based electrocatalysts have attracted considerable attention for HER owing to excellent conductivity, unique morphology, and a large specific surface which exhibits a great prospect for scale-up hydrogen production. In this article, research progress on the preparation of micro/nanofiber-based HER electrocatalysts by electrospinning technology is reviewed. Firstly, the characteristics of potential high-performance electrocatalysts for HER are elucidated. Secondly, the advantages of utilizing electrospinning technology for the preparation of electrocatalysts are summarized. Then, different types of electrospun micro/nanofiber-based HER electrocatalysts are discussed, including metal-based electrospun electrocatalyst (noble metals and alloys, transition metals and alloys), metal–non-metal electrocatalysts (metal sulfide-based electrocatalyst, metal oxide-based electrocatalysts, metal phosphide-based electrocatalysts, metal nitride-based electrocatalysts, metal carbide-based electrocatalysts), metal-free electrospun micro/nanofiber-based electrocatalysts, and hybrid electrospun micro/nanofiber-based electrocatalysts. Subsequently, the enhancement strategies for electrospun micro/nanofiber-based electrocatalysts are discussed. Despite the significant advances in the preparation of HER electrocatalysts by electrospinning has been obtained, various challenges in further improving their electrocatalytic performance and even practical applications need to be addressed:

(1) Optimization of electrospun micro/nanofiber-based electrocatalyst structures. The morphology and structure of electrospun micro/nanofibers play an important role in the performance of HER electrocatalysts. Numerous studies have shown that the specific surface area and pore size structure of electrospun micro/nanofiber-based electrocatalysts have an important influence on the exposure of active sites and mass transfer. Therefore, it is necessary to rationally design the structure of electrospun micro/nanofiber-based electrocatalysts (e.g., hollow structure, core-shell structure, and porous structure) to enhance their electrocatalytic capabilities. How to design and develop more delicate and novel structures of electrospun micro/nanofiber-based electrocatalysts to promote their performance is a worthy direction to study in the future.

(2) Development of self-supported electrospun micro/nanofiber-based electrocatalysts. Generally, a binder needs to be introduced to provide mechanical stability of the HER electrocatalyst for industrial applications, which hinders the mass transfer and the full exposure of the electrocatalytic active sites, stopping its further advancements. The literature has demonstrated that electrospun micro/nanofibers after high-temperature calcination or other heat treatments can be self-supported. However, they are still too fragile, resulting in a low structural stability. Therefore, the design and preparation of electrospun micro/nanofiber-based electrocatalysts with strong flexible and self-supported features is a key focus for future development.

(3) Design single-atom electrospun micro/nanofiber-based electrocatalysts. Electrospun micro/nanofiber-based electrocatalysts involve complex electronic structure changes as well as interfacial effects during the reaction process. In order to deeply reveal the effects of interfacial effects and electron transfer on the intermediates during the HER process, it is necessary to deeply explore the mechanisms affecting electrocatalytic activity at the atomic level. To address this issue, the design and development of single-atom electrospun micro/nanofiber-based electrocatalysts to realize the improvement of electrocatalytic performance is a promising method.

(4) Innovation of electrospun micro/nanofiber-based electrocatalysts with large pH adaptability. The pH value of the medium plays a crucial role in the electrocatalytic performance of electrospun micro/nanofiber-based electrocatalysts. To expand the application range of electrospun micro/nanofiber-based electrocatalysts, development of new kinds of electrocatalysts in the medium with a large range of pH without affecting the electrocatalytic activity is a challenging research direction for the future.

(5) Exploration of a reaction mechanism for electrospun fiber-based electrocatalysts in HER. To deeply understand the reaction mechanism of electrospun HER electrocatalysts, theoretical calculations (e.g., DFT) are widely used in analyzing the electrocatalytic reaction process. In the process of electrospun micro/nanofiber-based electrocatalyst performance enhancement, it may be accompanied by the discovery of new mechanisms. Therefore, it is necessary to combine theoretical calculations with advanced characterization tools (e.g., Operando Raman spectroscopy, in situ X-ray diffraction, etc.) for future study.

## Figures and Tables

**Table 1:** The parameters of the electrospinning process and their influences on morphology of micro/nanofiber.

Influence Factors	Electrospinning Parameters	Variations of Micro/Nanofiber Diameter and Morphology	Ref.
	Molecular Weight (↑)	Diameter (↑); Smooth surface	[69]
	Concentration (↑)	Diameter (↑); Beaded fibers	[70]
Solution Properties	Surface Tension (↑)	Diameter (↓); Beaded fibers	[71]
	Conductivity (↑)	Diameter (↓); Beaded fibers	[72,73,74]
	Applied Voltage (↑)	Diameter (↓) within a specific range	[75,76]
Processing Parameters	Feed Flow Rate (↑)	Diameter (↑); Beaded fibers	[77,78]
	Collector Distance (↑)	Diameter (↓); Difficult to collect	[79,80]
Environmental Conditions	Temperature (↑)	Diameter (↓)	[81]
	Humidity (↑)	Fiber of porous structure	[81,82,83]

↑: increase, ↓: decrease.

## Data Availability

Not applicable.

## References

[1] Cook T.R., Dogutan D.K., Reece S.Y., Surendranath Y., Teets T.S., Nocera D.G. (2010). Solar Energy Supply and Storage for the Legacy and Nonlegacy Worlds. Chem. Rev..

[2] Li L., Lin J., Wu N., Xie S., Meng C., Zheng Y., Wang X., Zhao Y. (2022). Review and outlook on the international renewable energy development. Energy Built Environ..

[3] Joo J., Kim T., Lee J., Choi S.I., Lee K. (2019). Morphology-Controlled Metal Sulfides and Phosphides for Electrochemical Water Splitting. Adv. Mater..

[4] Yu J., Le T.A., Tran N.Q., Lee H. (2020). Earth-Abundant Transition-Metal-Based Bifunctional Electrocatalysts for Overall Water Splitting in Alkaline Media. Chem. A Eur. J..

[5] Hassan Q., Viktor P., Al-Musawi T.J., Mahmood Ali B., Algburi S., Alzoubi H.M., Khudhair Al-Jiboory A., Zuhair Sameen A., Salman H.M., Jaszczur M. (2024). The renewable energy role in the global energy Transformations. Renew. Energy Focus.

[6] Jia Y., Jiang K., Wang H., Yao X. (2019). The Role of Defect Sites in Nanomaterials for Electrocatalytic Energy Conversion. Chem.

[7] Wang J., Xu F., Jin H., Chen Y., Wang Y. (2017). Non-Noble Metal-based Carbon Composites in Hydrogen Evolution Reaction: Fundamentals to Applications. Adv. Mater..

[8] Cui W., Cheng N., Liu Q., Ge C., Asiri A.M., Sun X. (2014). Mo_2_C Nanoparticles Decorated Graphitic Carbon Sheets: Biopolymer-Derived Solid-State Synthesis and Application as an Efficient Electrocatalyst for Hydrogen Generation. ACS Catal..

[9] Tian J., Liu Q., Asiri A.M., Sun X. (2014). Self-Supported Nanoporous Cobalt Phosphide Nanowire Arrays: An Efficient 3D Hydrogen-Evolving Cathode over the Wide Range of pH 0–14. J. Am. Chem. Soc..

[10] Zou X., Zhang Y. (2015). Noble metal-free hydrogen evolution catalysts for water splitting. Chem. Soc. Rev..

[11] Mahmood N., Yao Y., Zhang J.-W., Pan L., Zhang X., Zou J.-J. (2018). Electrocatalysts for Hydrogen Evolution in Alkaline Electrolytes: Mechanisms, Challenges, and Prospective Solutions. Adv. Sci..

[12] Cai J., Ding J., Wei D., Xie X., Li B., Lu S., Zhang J., Liu Y., Cai Q., Zang S. (2021). Coupling of Ru and O-Vacancy on 2D Mo-Based Electrocatalyst Via a Solid-Phase Interface Reaction Strategy for Hydrogen Evolution Reaction. Adv. Energy Mater..

[13] Pham T.A., Ping Y., Galli G. (2017). Modelling heterogeneous interfaces for solar water splitting. Nat. Mater..

[14] Dou S., Wang X., Wang S. (2019). Rational Design of Transition Metal-Based Materials for Highly Efficient Electrocatalysis. Small Methods.

[15] Kong D., Cha J.J., Wang H., Lee H.R., Cui Y. (2013). First-row transition metal dichalcogenide catalysts for hydrogen evolution reaction. Energy Environ. Sci..

[16] Megía P.J., Vizcaíno A.J., Calles J.A., Carrero A. (2021). Hydrogen Production Technologies: From Fossil Fuels toward Renewable Sources. A Mini Review. Energy Fuels.

[17] Jiang W.-J., Tang T., Zhang Y., Hu J.-S. (2020). Synergistic Modulation of Non-Precious-Metal Electrocatalysts for Advanced Water Splitting. Acc. Chem. Res..

[18] An L., Wei C., Lu M., Liu H., Chen Y., Scherer G.G., Fisher A.C., Xi P., Xu Z.J., Yan C.-H. (2021). Recent Development of Oxygen Evolution Electrocatalysts in Acidic Environment. Adv. Mater..

[19] Shi Y., Zhang B. (2016). Recent advances in transition metal phosphide nanomaterials: Synthesis and applications in hydrogen evolution reaction. Chem. Soc. Rev..

[20] Zeng K., Zhang D. (2010). Recent progress in alkaline water electrolysis for hydrogen production and applications. Prog. Energy Combust. Sci..

[21] Xu Y., Wang C., Huang Y., Fu J. (2021). Recent advances in electrocatalysts for neutral and large-current-density water electrolysis. Nano Energy.

[22] Younas M., Shafique S., Hafeez A., Javed F., Rehman F. (2022). An Overview of Hydrogen Production: Current Status, Potential, and Challenges. Fuel.

[23] Li G., Han G., Wang L., Cui X., Moehring N.K., Kidambi P.R., Jiang D.-e., Sun Y. (2023). Dual hydrogen production from electrocatalytic water reduction coupled with formaldehyde oxidation via a copper-silver electrocatalyst. Nat. Commun..

[24] Wei X., Zhang Y., He H., Gao D., Hu J., Peng H., Peng L., Xiao S., Xiao P. (2019). Carbon-incorporated NiO/Co_3_O_4_ concave surface microcubes derived from a MOF precursor for overall water splitting. Chem. Commun..

[25] Shan X., Liu J., Mu H., Xiao Y., Mei B., Liu W., Lin G., Jiang Z., Wen L., Jiang L. (2020). An Engineered Superhydrophilic/Superaerophobic Electrocatalyst Composed of the Supported CoMoS Chalcogel for Overall Water Splitting. Angew. Chem. Int. Ed..

[26] Zheng T., Shang C., He Z., Wang X., Cao C., Li H., Si R., Pan B., Zhou S., Zeng J. (2019). Intercalated Iridium Diselenide Electrocatalysts for Efficient pH-Universal Water Splitting. Angew. Chem. Int. Ed..

[27] Wang P., Jia T., Wang B. (2020). A critical review: 1D/2D nanostructured self-supported electrodes for electrochemical water splitting. J. Power Sources.

[28] Wang J., Hu J., Niu S., Li S., Du Y., Xu P. (2022). Crystalline-Amorphous Ni_2_P_4_O_12_/NiMoO Nanoarrays for Alkaline Water Electrolysis: Enhanced Catalytic Activity via In Situ Surface Reconstruction. Small.

[29] Lin L., Zhou W., Gao R., Yao S., Zhang X., Xu W., Zheng S., Jiang Z., Yu Q., Li Y.-W. (2017). Low-temperature hydrogen production from water and methanol using Pt/α-MoC catalysts. Nature.

[30] Tian J., Xu Z.-Y., Zhang D.-W., Wang H., Xie S.-H., Xu D.-W., Ren Y.-H., Wang H., Liu Y., Li Z.-T. (2016). Supramolecular metal-organic frameworks that display high homogeneous and heterogeneous photocatalytic activity for H_2_ production. Nat. Commun..

[31] Wang K., Huang B., Lin F., Lv F., Luo M., Zhou P., Liu Q., Zhang W., Yang C., Tang Y. (2018). Wrinkled Rh2P Nanosheets as Superior pH-Universal Electrocatalysts for Hydrogen Evolution Catalysis. Adv. Energy Mater..

[32] Hou J., Wu Y., Zhang B., Cao S., Li Z., Sun L. (2019). Rational Design of Nanoarray Architectures for Electrocatalytic Water Splitting. Adv. Funct. Mater..

[33] Liang C., Zou P., Nairan A., Zhang Y., Liu J., Liu K., Hu S., Kang F., Fan H.J., Yang C. (2020). Exceptional performance of hierarchical Ni–Fe oxyhydroxide@NiFe alloy nanowire array electrocatalysts for large current density water splitting. Energy Environ. Sci..

[34] He T., Peng Y., Li Q., Lu J.E., Liu Q., Mercado R., Chen Y., Nichols F., Zhang Y., Chen S. (2019). Nanocomposites Based on Ruthenium Nanoparticles Supported on Cobalt and Nitrogen-Codoped Graphene Nanosheets as Bifunctional Catalysts for Electrochemical Water Splitting. ACS Appl. Mater. Interfaces.

[35] Huang Z.-F., Wang J., Peng Y., Jung C.-Y., Fisher A., Wang X. (2017). Design of Efficient Bifunctional Oxygen Reduction/Evolution Electrocatalyst: Recent Advances and Perspectives. Adv. Energy Mater..

[36] Zhang X., Li J., Yang Y., Zhang S., Zhu H., Zhu X., Xing H., Zhang Y., Huang B., Guo S. (2018). Co_3_O_4_/Fe_0.33_Co_0.66_P Interface Nanowire for Enhancing Water Oxidation Catalysis at High Current Density. Adv. Mater..

[37] Dinh K.N., Liang Q., Du C.-F., Zhao J., Tok A.I.Y., Mao H., Yan Q. (2019). Nanostructured metallic transition metal carbides, nitrides, phosphides, and borides for energy storage and conversion. Nano Today.

[38] Gao R., Yan D. (2020). Recent Development of Ni/Fe-Based Micro/Nanostructures toward Photo/Electrochemical Water Oxidation. Adv. Energy Mater..

[39] Yan Y., Xia B.Y., Zhao B., Wang X. (2016). A review on noble-metal-free bifunctional heterogeneous catalysts for overall electrochemical water splitting. J. Mater. Chem. A.

[40] Yan X.-H., Prabhu P., Xu H., Meng Z., Xue T., Lee J.-M. (2020). Recent Progress of Metal Carbides Encapsulated in Carbon-Based Materials for Electrocatalysis of Oxygen Reduction Reaction. Small Methods.

[41] Prabhu P., Jose V., Lee J.-M. (2020). Heterostructured Catalysts for Electrocatalytic and Photocatalytic Carbon Dioxide Reduction. Adv. Funct. Mater..

[42] Xia Y., Yang P., Sun Y., Wu Y., Mayers B., Gates B., Yin Y., Kim F., Yan H. (2003). One-Dimensional Nanostructures: Synthesis, Characterization, and Applications. Adv. Mater..

[43] Yuan J., Xu Y., Müller A.H.E. (2011). One-dimensional magnetic inorganic–organic hybrid nanomaterials. Chem. Soc. Rev..

[44] Lu X., Zhang W., Wang C., Wen T.-C., Wei Y. (2011). One-dimensional conducting polymer nanocomposites: Synthesis, properties and applications. Prog. Polym. Sci..

[45] Li M., Zhu Y., Song N., Wang C., Lu X. (2018). Fabrication of Pt nanoparticles on nitrogen-doped carbon/Ni nanofibers for improved hydrogen evolution activity. J. Colloid Interface Sci..

[46] Huang Y., Miao Y.-E., Zhang L., Tjiu W.W., Pan J., Liu T. (2014). Synthesis of few-layered MoS_2_ nanosheet-coated electrospun SnO_2_ nanotube heterostructures for enhanced hydrogen evolution reaction. Nanoscale.

[47] Li Z., Zhang J.T., Chen Y.M., Li J., Lou X.W. (2015). Pie-like electrode design for high-energy density lithium–sulfur batteries. Nat. Commun..

[48] Ibrahim K.B., Tsai M.-C., Chala S.A., Berihun M.K., Kahsay A.W., Berhe T.A., Su W.-N., Hwang B.-J. (2019). A review of transition metal-based bifunctional oxygen electrocatalysts. J. Chin. Chem. Soc..

[49] Lyu F., Wang Q., Choi S.M., Yin Y. (2019). Noble-Metal-Free Electrocatalysts for Oxygen Evolution. Small.

[50] Yu F., Yu L., Mishra I.K., Yu Y., Ren Z.F., Zhou H.Q. (2018). Recent developments in earth-abundant and non-noble electrocatalysts for water electrolysis. Mater. Today Phys..

[51] Wang Y., Kong B., Zhao D., Wang H., Selomulya C. (2017). Strategies for developing transition metal phosphides as heterogeneous electrocatalysts for water splitting. Nano Today.

[52] Zeng M., Li Y. (2015). Recent advances in heterogeneous electrocatalysts for the hydrogen evolution reaction. J. Mater. Chem. A.

[53] Li W., Guo Z., Yang J., Li Y., Sun X., He H., Li S., Zhang J. (2022). Advanced Strategies for Stabilizing Single-Atom Catalysts for Energy Storage and Conversion. Electrochem. Energy Rev..

[54] Yusuf B.A., Yaseen W., Xie M., Zayyan R.S., Muhammad A.I., Nankya R., Xie J., Xu Y. (2023). Recent advances in understanding and design of efficient hydrogen evolution electrocatalysts for water splitting: A comprehensive review. Adv. Colloid Interface Sci..

[55] Quan L., Jiang H., Mei G., Sun Y., You B. (2024). Bifunctional Electrocatalysts for Overall and Hybrid Water Splitting. Chem. Rev..

[56] Zhang Z., Wu X., Kou Z., Song N., Nie G., Wang C., Verpoort F., Mu S. (2022). Rational design of electrospun nanofiber-typed electrocatalysts for water splitting: A review. Chem. Eng. J..

[57] Zhu J., Yan C., Li G., Cheng H., Li Y., Liu T., Mao Q., Cho H., Gao Q., Gao C. (2024). Recent developments of electrospun nanofibers for electrochemical energy storage and conversion. Energy Storage Mater..

[58] Lu X., Wang C., Favier F., Pinna N. (2017). Electrospun Nanomaterials for Supercapacitor Electrodes: Designed Architectures and Electrochemical Performance. Adv. Energy Mater..

[59] Nie G., Zhao X., Luan Y., Jiang J., Kou Z., Wang J. (2020). Key issues facing electrospun carbon nanofibers in energy applications: On-going approaches and challenges. Nanoscale.

[60] Wang X.-X., Yu G.-F., Zhang J., Yu M., Ramakrishna S., Long Y.-Z. (2021). Conductive polymer ultrafine fibers via electrospinning: Preparation, physical properties and applications. Prog. Mater. Sci..

[61] Han T., Reneker D.H., Yarin A.L. (2007). Buckling of jets in electrospinning. Polymer.

[62] Reneker D.H., Yarin A.L. (2008). Electrospinning jets and polymer nanofibers. Polymer.

[63] Li L., Peng S., Lee J.K.Y., Ji D., Srinivasan M., Ramakrishna S. (2017). Electrospun hollow nanofibers for advanced secondary batteries. Nano Energy.

[64] Qin Z., Yan G., Zhang X., Yang Z., Li H., Wang J. (2022). Finite element method assisted design of needleless electrospinning systems for mass production of polymer nanofibers. Chem. Eng. Sci..

[65] El-Sayed H., Vineis C., Varesano A., Mowafi S., Carletto R.A., Tonetti C., Taleb M.A. (2019). A critique on multi-jet electrospinning: State of the art and future outlook. Nanotechnol. Rev..

[66] Sun Z., Zussman E., Yarin A.L., Wendorff J.H., Greiner A. (2003). Compound Core–Shell Polymer Nanofibers by Co-Electrospinning. Adv. Mater..

[67] Gao Q., Agarwal S., Greiner A., Zhang T. (2023). Electrospun fiber-based flexible electronics: Fiber fabrication, device platform, functionality integration and applications. Prog. Mater. Sci..

[68] Doshi J., Reneker D.H. (1995). Electrospinning process and applications of electrospun fibers. J. Electrost..

[69] Koski A., Yim K., Shivkumar S. (2004). Effect of molecular weight on fibrous PVA produced by electrospinning. Mater. Lett..

[70] Zhang J., Yu M., Tao S. (2024). Advanced electrospinning nanomaterials: From spinning fabrication techniques to electrochemical applications. Nano Res..

[71] Fong H., Chun I., Reneker D.H. (1999). Beaded nanofibers formed during electrospinning. Polymer.

[72] Matabola K.P., Moutloali R.M. (2013). The influence of electrospinning parameters on the morphology and diameter of poly(vinyledene fluoride) nanofibers- effect of sodium chloride. J. Mater. Sci..

[73] Mokhtari F., Shamshirsaz M., Latifi M. (2016). Investigation of β phase formation in piezoelectric response of electrospun polyvinylidene fluoride nanofibers: LiCl additive and increasing fibers tension. Polym. Eng. Sci..

[74] Shokrollahzadeh S., Tajik S. (2018). Fabrication of thin film composite forward osmosis membrane using electrospun polysulfone/polyacrylonitrile blend nanofibers as porous substrate. Desalination.

[75] Zong X., Kim K., Fang D., Ran S., Hsiao B.S., Chu B. (2002). Structure and process relationship of electrospun bioabsorbable nanofiber membranes. Polymer.

[76] Hu J., Wang X., Ding B., Lin J., Yu J., Sun G. (2011). One-step Electro-spinning/netting Technique for Controllably Preparing Polyurethane Nano-fiber/net. Macromol. Rapid Commun..

[77] Asmatulu R., Veisi Z., Uddin M.N., Mahapatro A. (2019). Highly Sensitive and Reliable Electrospun Polyaniline Nanofiber Based Biosensor as a Robust Platform for COX-2 Enzyme Detections. Fibers Polym..

[78] Topuz F., Abdulhamid M.A., Holtzl T., Szekely G. (2021). Nanofiber engineering of microporous polyimides through electrospinning: Influence of electrospinning parameters and salt addition. Mater. Des..

[79] Xue J., Wu T., Dai Y., Xia Y. (2019). Electrospinning and Electrospun Nanofibers: Methods, Materials, and Applications. Chem. Rev..

[80] Zhang X., Lu Y. (2014). Centrifugal Spinning: An Alternative Approach to Fabricate Nanofibers at High Speed and Low Cost. Polym. Rev..

[81] Mit-uppatham C., Nithitanakul M., Supaphol P. (2004). Ultrafine Electrospun Polyamide-6 Fibers: Effect of Solution Conditions on Morphology and Average Fiber Diameter. Macromol. Chem. Phys..

[82] Casper C.L., Stephens J.S., Tassi N.G., Chase D.B., Rabolt J.F. (2004). Controlling Surface Morphology of Electrospun Polystyrene Fibers:  Effect of Humidity and Molecular Weight in the Electrospinning Process. Macromolecules.

[83] Li D., Xia Y. (2004). Electrospinning of Nanofibers: Reinventing the Wheel?. Adv. Mater..

[84] Ramasamy E., Lee W.J., Lee D.Y., Song J.S. (2007). Nanocarbon counterelectrode for dye sensitized solar cells. Appl. Phys. Lett..

[85] Yu X., Ye S. (2007). Recent advances in activity and durability enhancement of Pt/C catalytic cathode in PEMFC: Part I. Physico-chemical and electronic interaction between Pt and carbon support, and activity enhancement of Pt/C catalyst. J. Power Sources.

[86] Koenigsmann C., Santulli A.C., Gong K., Vukmirovic M.B., Zhou W.-p., Sutter E., Wong S.S., Adzic R.R. (2011). Enhanced Electrocatalytic Performance of Processed, Ultrathin, Supported Pd–Pt Core–Shell Nanowire Catalysts for the Oxygen Reduction Reaction. J. Am. Chem. Soc..

[87] Zheng Y., Jiao Y., Qiao S.Z. (2015). Engineering of Carbon-Based Electrocatalysts for Emerging Energy Conversion: From Fundamentality to Functionality. Adv. Mater..

[88] Chen X., Niu K., Xue Z., Liu X., Liu B., Zhang B., Zeng H., Lv W., Zhang Y., Wu Y. (2022). Ultrafine platinum nanoparticles supported on N,S-codoped porous carbon nanofibers as efficient multifunctional materials for noticeable oxygen reduction reaction and water splitting performance. Nanoscale Adv..

[89] Wang X., Zhang Y., Li J., Liu G., Gao M., Ren S., Liu B., Zhang L., Han G., Yu J. (2022). Platinum Cluster/Carbon Quantum Dots Derived Graphene Heterostructured Carbon Nanofibers for Efficient and Durable Solar-Driven Electrochemical Hydrogen Evolution. Small Methods.

[90] Zhang Z., Cai J., Zhu H., Zhuang Z., Xu F., Hao J., Lu S., Li H., Duan F., Du M. (2020). Simple construction of ruthenium single atoms on electrospun nanofibers for superior alkaline hydrogen evolution: A dynamic transformation from clusters to single atoms. Chem. Eng. J..

[91] He R., Yang Y., Yang P., Zhao X., Zhu J., Yang R., Huang Q., Yang L. (2022). Electrospun nano-Ir anchored mesoporous carbon nanofibers for hydrogen evolution reaction. Chem. Phys..

[92] Zhang M., Wang S., Li T., Chen J., Zhu H., Du M. (2016). Nitrogen and gold nanoparticles co-doped carbon nanofiber hierarchical structures for efficient hydrogen evolution reactions. Electrochim. Acta.

[93] Li M., Yu J., Liu Q., Liu J., Chen R., Zhu J., Li R., Wang J. (2022). Atomically Dispersed Fe–N5 Sites Anchored in Porous N-Doped Carbon Nanofibers for Effective Hydrogen Evolution Reaction. ACS Sustain. Chem. Eng..

[94] Wang Z., Zuo P., Fan L., Han J., Xiong Y., Yin G. (2016). Facile electrospinning preparation of phosphorus and nitrogen dual-doped cobalt-based carbon nanofibers as bifunctional electrocatalyst. J. Power Sources.

[95] Zhao Y., Zhang J., Li K., Ao Z., Wang C., Liu H., Sun K., Wang G. (2016). Electrospun cobalt embedded porous nitrogen doped carbon nanofibers as an efficient catalyst for water splitting. J. Mater. Chem. A.

[96] Mukhiya T., Muthurasu A., Tiwari A.P., Chhetri K., Chae S.-H., Kim H., Dahal B., Lee B.M., Kim H.Y. (2021). Integrating the Essence of a Metal–Organic Framework with Electrospinning: A New Approach for Making a Metal Nanoparticle Confined N-Doped Carbon Nanotubes/Porous Carbon Nanofibrous Membrane for Energy Storage and Conversion. ACS Appl. Mater. Interfaces.

[97] Ding Q., Liu M., Miao Y.-E., Huang Y., Liu T. (2015). Electrospun nickel-decorated carbon nanofiber membranes as efficient electrocatalysts for hydrogen evolution reaction. Electrochim. Acta.

[98] Wang J., Zhu H., Chen J., Zhang B., Zhang M., Wang L., Du M. (2016). Small and well-dispersed Cu nanoparticles on carbon nanofibers: Self-supported electrode materials for efficient hydrogen evolution reaction. Int. J. Hydrogen Energy.

[99] Li L., Gu J., Ye Y., Guo J., Zhao J., Zou G. (2022). Effective synergy between palladium nanoparticles and nitrogen-doped porous carbon fiber for hydrogen evolution reaction. Electrochim. Acta.

[100] Guan J., Chen W., Zhu Y., Wang L., Fu Y., Guo B., Zhang M. (2023). Integrating RuCo alloy in N-doped carbon nanofiber for efficient hydrogen evolution in alkaline media. J. Alloys Compd..

[101] Kwon T., Yu A., Kim S.-j., Kim M.H., Lee C., Lee Y. (2021). Au-Ru alloy nanofibers as a highly stable and active bifunctional electrocatalyst for acidic water splitting. Appl. Surf. Sci..

[102] Kim Y., Yu A., Lee Y. (2021). Iridium-cobalt alloy nanotubes as a bifunctional electrocatalyst for pH-universal overall water splitting. Bull. Korean Chem. Soc..

[103] Jin D., Lee Y., Hwa Kim M., Lee C. (2021). A fascinating pH independent catalyst for hydrogen evolution reaction: Crystalline bimetallic hcp-CoxRh1-x alloy nanofibers driven by thermally induced phase transition from a single phase of CoRh_2_O_4_. Appl. Surf. Sci..

[104] Nam Y., Jin D., Lee C., Lee Y. (2023). Fe-Cu-Rh ternary alloy nanofibers as an outstanding pH-universal electrocatalyst for hydrogen evolution reaction: The catalytic roles of Fe depending on pH. Appl. Surf. Sci..

[105] Zhang B., Zhu H., Zou M., Liu X., Yang H., Zhang M., Wu W., Yao J., Du M. (2017). Design and fabrication of size-controlled Pt–Au bimetallic alloy nanostructure in carbon nanofibers: A bifunctional material for biosensors and the hydrogen evolution reaction. J. Mater. Sci..

[106] Zhang J., Xia Z., Dai L. (2015). Carbon-based electrocatalysts for advanced energy conversion and storage. Sci. Adv..

[107] Shanmugapriya S., Zhu P., Yan C., Asiri A.M., Zhang X., Selvan R.K. (2019). Multifunctional High-Performance Electrocatalytic Properties of Nb2O5 Incorporated Carbon Nanofibers as Pt Support Catalyst. Adv. Mater. Interfaces.

[108] Shanmugapriya S., Zhu P., Ganeshbabu M., Sung Lee Y., Zhang X., Kalai Selvan R. (2022). Improved electrocatalytic properties of bundled B/N co-doped electrospun carbon nanofibers with Pt nanostructures through dopant-induced metal-support interaction (DIMSI). Mater. Sci. Eng. B.

[109] Liu X., Zhang M., Yang T., Wang L., Zhu H., Wang S., Du M. (2016). Carbon nanofibers as nanoreactors in the construction of PtCo alloy carbon core-shell structures for highly efficient and stable water splitting. Mater. Des..

[110] Wang P., Jiang K., Wang G., Yao J., Huang X. (2016). Phase and Interface Engineering of Platinum–Nickel Nanowires for Efficient Electrochemical Hydrogen Evolution. Angew. Chem. Int. Ed..

[111] Zhai H., Xu G., Liu J., Xu T., Li C., Bai J. (2022). Boosting activity and stability by coupling Pt–Ni nanoparticles with La-modified flexible carbon nanofibers for hydrogen evolution reaction. Int. J. Hydrogen Energy.

[112] Wang J., Chen J.W., Chen J.D., Zhu H., Zhang M., Du M.L. (2017). Designed Synthesis of Size-Controlled Pt—Cu Alloy Nanoparticles Encapsulated in Carbon Nanofibers and Their High Efficient Electrocatalytic Activity Toward Hydrogen Evolution Reaction. Adv. Mater. Interfaces.

[113] Zhang J., Jia W., Dang S., Cao Y. (2020). Sub-5 nm octahedral platinum-copper nanostructures anchored on nitrogen-doped porous carbon nanofibers for remarkable electrocatalytic hydrogen evolution. J. Colloid Interface Sci..

[114] Han Y., Duan H., Liu W., Zhou C., Wang B., Jiang Q., Feng S., Yan W., Tan T., Zhang R. (2023). Engineering the electronic structure of platinum single-atom sites via tailored porous carbon nanofibers for large-scale hydrogen production. Appl. Catal. B Environ..

[115] Li M., Wang H., Zhu W., Li W., Wang C., Lu X. (2020). RuNi Nanoparticles Embedded in N-Doped Carbon Nanofibers as a Robust Bifunctional Catalyst for Efficient Overall Water Splitting. Adv. Sci..

[116] Chen X., Yan S., Wen S., Chen J., Xu J., Wang C., Lu X. (2023). Chelating adsorption-engaged synthesis of ultrafine iridium nanoparticles anchored on N-doped carbon nanofibers toward highly efficient hydrogen evolution in both alkaline and acidic media. J. Colloid Interface Sci..

[117] Chen X., Gou W., Xu J., Qi R., Ren S., Wang C., Chen W., Yu G., Lu X. (2023). Low-loading and ultrasmall Ir nanoparticles coupled with Ni/nitrogen-doped carbon nanofibers with Pt-like hydrogen evolution performance in both acidic and alkaline media. Chem. Eng. J..

[118] Chen J., Chen J., Yu D., Zhang M., Zhu H., Du M. (2017). Carbon nanofiber-supported PdNi alloy nanoparticles as highly efficient bifunctional catalysts for hydrogen and oxygen evolution reactions. Electrochim. Acta.

[119] Quaino P., Juarez F., Santos E., Schmickler W. (2014). Volcano plots in hydrogen electrocatalysis—Uses and abuses. Beilstein J. Nanotechnol..

[120] Zhu L., Lin H., Li Y., Liao F., Lifshitz Y., Sheng M., Lee S.-T., Shao M. (2016). A rhodium/silicon co-electrocatalyst design concept to surpass platinum hydrogen evolution activity at high overpotentials. Nat. Commun..

[121] Yu A., Kim S.Y., Lee C., Kim M.H., Lee Y. (2019). Boosted Electron-Transfer Kinetics of Hydrogen Evolution Reaction at Bimetallic RhCo Alloy Nanotubes in Acidic Solution. ACS Appl. Mater. Interfaces.

[122] Wang J., Zhu H., Yu D., Chen J., Chen J., Zhang M., Wang L., Du M. (2017). Engineering the Composition and Structure of Bimetallic Au–Cu Alloy Nanoparticles in Carbon Nanofibers: Self-Supported Electrode Materials for Electrocatalytic Water Splitting. ACS Appl. Mater. Interfaces.

[123] Alali K.T., Jing y., Moharram D., Liu Q., Chen R., Zhu J., Li R., Liu P., Liu J., Wang J. (2021). In situ construction of 3-dimensional hierarchical carbon nanostructure; investigation of the synthesis parameters and hydrogen evolution reaction performance. Carbon.

[124] Wang S., Wang J., Wang X., Li L., Qin J., Cao M. (2021). Carbon hybrid with 3D nano-forest architecture in-situ catalytically constructed by CoFe alloy as advanced multifunctional electrocatalysts for Zn-air batteries-driven water splitting. J. Energy Chem..

[125] Cardoso D.S.P., Amaral L., Santos D.M.F., Šljukić B., Sequeira C.A.C., Macciò D., Saccone A. (2015). Enhancement of hydrogen evolution in alkaline water electrolysis by using nickel-rare earth alloys. Int. J. Hydrogen Energy.

[126] Li T., Li S., Liu Q., Yin J., Sun D., Zhang M., Xu L., Tang Y., Zhang Y. (2020). Immobilization of Ni3Co Nanoparticles into N-Doped Carbon Nanotube/Nanofiber Integrated Hierarchically Branched Architectures toward Efficient Overall Water Splitting. Adv. Sci..

[127] Zhang B., Shan J., Yu J., Wang W., Li W., Li N., Li Y. (2021). Electrospun prussian blue analogue derived NiCo@N-doped carbon nanofibers as efficient and highly stable electrocatalysts for neutral overall water splitting. Int. J. Hydrogen Energy.

[128] Li C., Liu J., Gao R., Ouyang H., Huang J., Huang Q., Liu Y. (2022). Tailoring CoNi alloy embedded carbon nano-fibers by thiourea for enhanced hydrogen evolution reaction. Int. J. Hydrogen Energy.

[129] Li M., Liu T., Bo X., Zhou M., Guo L. (2017). A novel flower-like architecture of FeCo@NC-functionalized ultra-thin carbon nanosheets as a highly efficient 3D bifunctional electrocatalyst for full water splitting. J. Mater. Chem. A.

[130] Aftab F., Duran H., Kirchhoff K., Zaheer M., Iqbal B., Saleem M., Arshad S.N. (2020). A Facile Synthesis of FeCo Nanoparticles Encapsulated in Hierarchical N-Doped Carbon Nanotube/Nanofiber Hybrids for Overall Water Splitting. ChemCatChem.

[131] Li T., Luo G., Liu K., Li X., Sun D., Xu L., Li Y., Tang Y. (2018). Encapsulation of Ni3Fe Nanoparticles in N-Doped Carbon Nanotube–Grafted Carbon Nanofibers as High-Efficiency Hydrogen Evolution Electrocatalysts. Adv. Funct. Mater..

[132] Sun M., Ye Q., Lin L., Wang Y., Zheng Z., Chen F., Cheng Y. (2023). NiMo solid-solution alloy porous nanofiber as outstanding hydrogen evolution electrocatalyst. J. Colloid Interface Sci..

[133] Yu S., Liu M., Wang Q., Xu J., Zou Y., Xiang C., Xu F., Sun L., Jiang M., Hu Z. (2023). Three-dimensional carbon nanofiber networks encapsulated in cobalt–molybdenum metal clusters on nitrogen-doped carbon as ultra-efficient electrocatalysts for hydrogen evolution reactions. J. Alloys Compd..

[134] Ghouri Z.K., Badreldin A., Elsaid K., Kumar D., Youssef K., Abdel-Wahab A. (2021). Theoretical and experimental investigations of Co-Cu bimetallic alloys-incorporated carbon nanowires as an efficient bi-functional electrocatalyst for water splitting. J. Ind. Eng. Chem..

[135] Guan J., Liu Y., Fang Y., Du X., Fu Y., Wang L., Zhang M. (2021). Co-Ni alloy nanoparticles supported by carbon nanofibers for hydrogen evolution reaction. J. Alloys Compd..

[136] Sankar S.S., Karthick K., Kumaravel S., Karmakar A., Ragunath M., Kundu S. (2021). Temperature-Controlled Structural Variations of Meticulous Fibrous Networks of NiFe-Polymeric Zeolite Imidazolate Frameworks for Enhanced Performance in Electrocatalytic Water-Splitting Reactions. Inorg. Chem..

[137] Yang H., Zhang T., Zhu H., Zhang M., Wu W., Du M. (2017). Synthesis of a MoS_2_(1−x)Se_2_x ternary alloy on carbon nanofibers as the high efficient water splitting electrocatalyst. Int. J. Hydrogen Energy.

[138] Heo E., Noh S., Jo H., Lee H., Lee S., Kim M., Lee J., Yoon H. (2021). Carbon Nanofibers Featuring Bimetallic Nanoparticle-in-Pore Structures as Water-Splitting Electrocatalysts. ACS Appl. Nano Mater..

[139] Zhu H., Du M., Zhang M., Zou M., Yang T., Wang S., Yao J., Guo B. (2014). S-rich single-layered MoS_2_ nanoplates embedded in N-doped carbon nanofibers: Efficient co-electrocatalysts for the hydrogen evolution reaction. Chem. Commun..

[140] Liu P., Li J., Lu Y., Xiang B. (2018). Facile synthesis of NiS_2_ nanowires and its efficient electrocatalytic performance for hydrogen evolution reaction. Int. J. Hydrogen Energy.

[141] Manjunatha C., Patil R.S., Sudeep M., Srinivasa N., Kumar R.C., Sham Aan M.P., Ashoka S. (2020). Rational design and synthesis of hetero-nanostructured electrospun PU@PANI@FeS2: A surface tailored hybrid catalyst for H_2_ production via electrochemical splitting of water. Surf. Interfaces.

[142] Zhu W., Cheng Y., Wang C., Pinna N., Lu X. (2021). Transition metal sulfides meet electrospinning: Versatile synthesis, distinct properties and prospective applications. Nanoscale.

[143] Yun Q., Li L., Hu Z., Lu Q., Chen B., Zhang H. (2020). Layered Transition Metal Dichalcogenide-Based Nanomaterials for Electrochemical Energy Storage. Adv. Mater..

[144] Gu H., Huang Y., Zuo L., Fan W., Liu T. (2016). Electrospun carbon nanofiber@CoS_2_ core/sheath hybrid as an efficient all-pH hydrogen evolution electrocatalyst. Inorg. Chem. Front..

[145] Zhang Z., Wang Y., Leng X., Crespi V.H., Kang F., Lv R. (2018). Controllable Edge Exposure of MoS_2_ for Efficient Hydrogen Evolution with High Current Density. ACS Appl. Energy Mater..

[146] Zhu H., Du M., Zhang M., Zou M., Yang T., Fu Y., Yao J. (2014). The design and construction of 3D rose-petal-shaped MoS_2_ hierarchical nanostructures with structure-sensitive properties. J. Mater. Chem. A.

[147] Zhang X., Li L., Guo Y., Liu D., You T. (2016). Amorphous flower-like molybdenum-sulfide-@-nitrogen-doped-carbon-nanofiber film for use in the hydrogen-evolution reaction. J. Colloid Interface Sci..

[148] Zhu X., Mo L., Wu Y., Lai F., Han X., Ling X.Y., Liu T., Miao Y.-E. (2018). Self-supported MoS_2_@NHCF fiber-in-tube composites with tunable voids for efficient hydrogen evolution reaction. Compos. Commun..

[149] Wu W., Zhao Y., Li S., He B., Liu H., Zeng X., Zhang J., Wang G. (2019). P doped MoS_2_ nanoplates embedded in nitrogen doped carbon nanofibers as an efficient catalyst for hydrogen evolution reaction. J. Colloid Interface Sci..

[150] Guan J., Chen W., Fang Y., Wang L., Fu Y., Guo B., Zhang M. (2022). Electrospinning derivative fabrication of sandwich-structured CNF/Co_3_S_4_/MoS_2_ as self-supported electrodes to accelerate electron transport in HER. Int. J. Hydrogen Energy.

[151] Yu S., Kim J., Yoon K.R., Jung J.-W., Oh J., Kim I.-D. (2015). Rational Design of Efficient Electrocatalysts for Hydrogen Evolution Reaction: Single Layers of WS_2_ Nanoplates Anchored to Hollow Nitrogen-Doped Carbon Nanofibers. ACS Appl. Mater. Interfaces.

[152] Liu Z., Li N., Su C., Zhao H., Xu L., Yin Z., Li J., Du Y. (2018). Colloidal synthesis of 1T’ phase dominated WS2 towards endurable electrocatalysis. Nano Energy.

[153] Zheng X., Xiao Y., Miao X., Wang Y., Chen Y., Hu T., Gong X., Wu C., Wang G., Liu H. (2022). Structure tailorable and flexible electrospun Poly(vinylidene fluoride)@WS_2_ membrane with enhanced hydrogen evolution reaction activity and mechanical properties. Polym. Test..

[154] Sun C., Zhang J., Ma J., Liu P., Gao D., Tao K., Xue D. (2016). N-doped WS_2_ nanosheets: A high-performance electrocatalyst for the hydrogen evolution reaction. J. Mater. Chem. A.

[155] Chakrapani V., Thangala J., Sunkara M.K. (2009). WO3 and W2N nanowire arrays for photoelectrochemical hydrogen production. Int. J. Hydrogen Energy.

[156] Wan M., Li J., Li T., Zhu H., Wu W., Du M. (2018). Nitrogen anion-decorated cobalt tungsten disulfides solid solutions on the carbon nanofibers for water splitting. Nanotechnology.

[157] Wan S., Liu Y., Li G.-D., Li X., Wang D., Zou X. (2016). Well-dispersed CoS_2_ nano-octahedra grown on a carbon fibre network as efficient electrocatalysts for hydrogen evolution reaction. Catal. Sci. Technol..

[158] Li Y., Ma W., Zeng Y., Chen X., Wang J., Zhong Q. (2022). A monolith electrode featuring FeS_2_ embedded in porous carbon nanofibers for efficient hydrogen evolution. Electrochim. Acta.

[159] Liu J., Wang J., Zhang B., Ruan Y., Lv L., Ji X., Xu K., Miao L., Jiang J. (2017). Hierarchical NiCo_2_S_4_@NiFe LDH Heterostructures Supported on Nickel Foam for Enhanced Overall-Water-Splitting Activity. ACS Appl. Mater. Interfaces.

[160] Zhou X., Zhou J., Huang G., Fan R., Ju S., Mi Z., Shen M. (2018). A bifunctional and stable Ni–Co–S/Ni–Co–P bistratal electrocatalyst for 10.8%-efficient overall solar water splitting. J. Mater. Chem. A.

[161] Xia C., Li P., Gandi A.N., Schwingenschlögl U., Alshareef H.N. (2015). Is NiCo2S4 Really a Semiconductor?. Chem. Mater..

[162] Ma L., Hu Y., Chen R., Zhu G., Chen T., Lv H., Wang Y., Liang J., Liu H., Yan C. (2016). Self-assembled ultrathin NiCo_2_S_4_ nanoflakes grown on Ni foam as high-performance flexible electrodes for hydrogen evolution reaction in alkaline solution. Nano Energy.

[163] Gu H., Fan W., Liu T. (2017). Phosphorus-doped NiCo2S4 nanocrystals grown on electrospun carbon nanofibers as ultra-efficient electrocatalysts for the hydrogen evolution reaction. Nanoscale Horiz..

[164] Yin X., Sun G., Wang L., Bai L., Su L., Wang Y., Du Q., Shao G. (2017). 3D hierarchical network NiCo_2_S_4_ nanoflakes grown on Ni foam as efficient bifunctional electrocatalysts for both hydrogen and oxygen evolution reaction in alkaline solution. Int. J. Hydrogen Energy.

[165] Zhou J., Dou Y., He T., Zhou A., Kong X.-J., Wu X.-Q., Liu T., Li J.-R. (2021). Revealing the effect of anion-tuning in bimetallic chalcogenides on electrocatalytic overall water splitting. Nano Res..

[166] Ranjith K.S., Kwak C.H., Ghoreishian S.M., Im J.S., Huh Y.S., Han Y.-K. (2020). Ultrathin rGO-wrapped free-standing bimetallic CoNi_2_S_4_-carbon nanofibers: An efficient and robust bifunctional electrocatalyst for water splitting. Nanotechnology.

[167] Yu S., Zou Y., Wang Q., Xu J., Xiang C., Xu F., Sun L., Yang F. (2022). Self-supported Co–Mo sulfide in electrospun carbon nanofibers as electrocatalysts for hydrogen evolution reaction in alkaline medium. J. Alloys Compd..

[168] Maduraiveeran G., Sasidharan M., Jin W. (2019). Earth-abundant transition metal and metal oxide nanomaterials: Synthesis and electrochemical applications. Prog. Mater. Sci..

[169] Hwang J., Rao R.R., Giordano L., Katayama Y., Yu Y., Shao-Horn Y. (2017). Perovskites in catalysis and electrocatalysis. Science.

[170] Xue Y., Sun S., Wang Q., Dong Z., Liu Z. (2018). Transition metal oxide-based oxygen reduction reaction electrocatalysts for energy conversion systems with aqueous electrolytes. J. Mater. Chem. A.

[171] Chen Z., Duan X., Wei W., Wang S., Ni B.-J. (2019). Recent advances in transition metal-based electrocatalysts for alkaline hydrogen evolution. J. Mater. Chem. A.

[172] Suen N.-T., Hung S.-F., Quan Q., Zhang N., Xu Y.-J., Chen H.M. (2017). Electrocatalysis for the oxygen evolution reaction: Recent development and future perspectives. Chem. Soc. Rev..

[173] Gong M., Zhou W., Tsai M.-C., Zhou J., Guan M., Lin M.-C., Zhang B., Hu Y., Wang D.-Y., Yang J. (2014). Nanoscale nickel oxide/nickel heterostructures for active hydrogen evolution electrocatalysis. Nat. Commun..

[174] Gong M., Wang D.-Y., Chen C.-C., Hwang B.-J., Dai H. (2016). A mini review on nickel-based electrocatalysts for alkaline hydrogen evolution reaction. Nano Res..

[175] Chen J., Yu D., Liao W., Zheng M., Xiao L., Zhu H., Zhang M., Du M., Yao J. (2016). WO_3_–x Nanoplates Grown on Carbon Nanofibers for an Efficient Electrocatalytic Hydrogen Evolution Reaction. ACS Appl. Mater. Interfaces.

[176] Cho Y.-B., Yu A., Lee C., Kim M.H., Lee Y. (2018). Fundamental Study of Facile and Stable Hydrogen Evolution Reaction at Electrospun Ir and Ru Mixed Oxide Nanofibers. ACS Appl. Mater. Interfaces.

[177] Zhu Y., Zhou W., Zhong Y., Bu Y., Chen X., Zhong Q., Liu M., Shao Z. (2017). A Perovskite Nanorod as Bifunctional Electrocatalyst for Overall Water Splitting. Adv. Energy Mater..

[178] Hua B., Li M., Zhang Y.-Q., Sun Y.-F., Luo J.-L. (2017). All-In-One Perovskite Catalyst: Smart Controls of Architecture and Composition toward Enhanced Oxygen/Hydrogen Evolution Reactions. Adv. Energy Mater..

[179] Zhang Z., He B., Chen L., Wang H., Wang R., Zhao L., Gong Y. (2018). Boosting Overall Water Splitting via FeOOH Nanoflake-Decorated PrBa0.5Sr0.5Co2O5+δ Nanorods. ACS Appl. Mater. Interfaces.

[180] He B., Tan K., Gong Y., Wang R., Wang H., Zhao L. (2020). Coupling amorphous cobalt hydroxide nanoflakes on Sr_2_Fe_1.5_Mo_0.5_O_5_+δ perovskite nanofibers to induce bifunctionality for water splitting. Nanoscale.

[181] Yu J., Zhou W., Xiong T., Wang A., Chen S., Chu B. (2017). Enhanced electrocatalytic activity of Co@N-doped carbon nanotubes by ultrasmall defect-rich TiO2 nanoparticles for hydrogen evolution reaction. Nano Res..

[182] Chinnappan A., Dongxiao J., Jayathilaka W.A.D.M., Baskar C., Qin X., Ramakrishna S. (2018). Facile synthesis of electrospun C@NiO/Ni nanofibers as an electrocatalyst for hydrogen evolution reaction. Int. J. Hydrogen Energy.

[183] Wang P., Zhang X., Wei Y., Yang P. (2019). Ni/NiO nanoparticles embedded inporous graphite nanofibers towards enhanced electrocatalytic performance. Int. J. Hydrogen Energy.

[184] Yang L., Zhao X., Yang R., Zhao P., Li Y., Yang P., Wang J., Astruc D. (2019). In-situ growth of carbon nanotubes on Ni/NiO nanofibers as efficient hydrogen evolution reaction catalysts in alkaline media. Appl. Surf. Sci..

[185] Surendran S., Jesudass S.C., Janani G., Kim J.Y., Lim Y., Park J., Han M.-K., Cho I.S., Sim U. (2023). Sulphur Assisted Nitrogen-Rich CNF for Improving Electronic Interactions in Co-NiO Heterostructures Toward Accelerated Overall Water Splitting. Adv. Mater. Technol..

[186] Zhong M., Yan S., Xu J., Wang C., Lu X. (2022). Manipulating Ru oxidation within electrospun carbon nanofibers to boost hydrogen and oxygen evolution for electrochemical overall water splitting. Inorg. Chem. Front..

[187] Anis S.F., Mostafa A.O., Hilal N., Hashaikeh R. (2020). Nanocrystalline NiWO_4_-WO_3_-WO_2.9_ Composite Strings: Fabrication, Characterization and their Electrocatalytic Performance for Hydrogen Evolution Reaction. Metall. Mater. Trans. A.

[188] Cui F., Tang L., Han W., Liu Y.-T., Kim H.Y., Yu J., Ding B. (2020). Novel synthesis of Al-amorphized, flexible Fe_2_O_3_ nanofibrous membranes for enhanced electrocatalytic H_2_ evolution. Compos. Commun..

[189] Navarro-Pardo F., Liu J., Abdelkarim O., Selopal G.S., Yurtsever A., Tavares A.C., Zhao H., Wang Z.M., Rosei F. (2020). 1D/2D Cobalt-Based Nanohybrids as Electrocatalysts for Hydrogen Generation. Adv. Funct. Mater..

[190] Chen X., Liu L., Yu P.Y., Mao S.S. (2011). Increasing Solar Absorption for Photocatalysis with Black Hydrogenated Titanium Dioxide Nanocrystals. Science.

[191] Xu Y., Zhang C., Zhang L., Zhang X., Yao H., Shi J. (2016). Pd-catalyzed instant hydrogenation of TiO_2_ with enhanced photocatalytic performance. Energy Environ. Sci..

[192] Yu J., Wang X., Huang X., Cao J., Huo Q., Mi L., Yang H., Hu Q., He C. (2022). Confining ultrafine Ru clusters into TiO_2_ lattice frameworks to yield efficient and ultrastable electrocatalysts towards practical hydrogen evolution. Chem. Eng. J..

[193] Hu Q., Wang Z., Huang X., Qin Y., Yang H., Ren X., Zhang Q., Liu J., He C. (2020). A unique space confined strategy to construct defective metal oxides within porous nanofibers for electrocatalysis. Energy Environ. Sci..

[194] Yang H., Liu Y., Luo S., Zhao Z., Wang X., Luo Y., Wang Z., Jin J., Ma J. (2017). Lateral-Size-Mediated Efficient Oxygen Evolution Reaction: Insights into the Atomically Thin Quantum Dot Structure of NiFe_2_O_4_. ACS Catal..

[195] Yue Q., Liu C., Wan Y., Wu X., Zhang X., Du P. (2018). Defect engineering of mesoporous nickel ferrite and its application for highly enhanced water oxidation catalysis. J. Catal..

[196] Selvasundarasekar S.S., Bijoy T.K., Kumaravel S., Karmakar A., Madhu R., Bera K., Nagappan S., Dhandapani H.N., Lee S.-C., Kundu S. (2022). Constructing electrospun spinel NiFe_2_O_4_ nanofibers decorated with palladium ions as nanosheets heterostructure: Boosting electrocatalytic activity of HER in alkaline water electrolysis. Nanoscale.

[197] Chen D., Chen C., Baiyee Z.M., Shao Z., Ciucci F. (2015). Nonstoichiometric Oxides as Low-Cost and Highly-Efficient Oxygen Reduction/Evolution Catalysts for Low-Temperature Electrochemical Devices. Chem. Rev..

[198] Hong W.T., Risch M., Stoerzinger K.A., Grimaud A., Suntivich J., Shao-Horn Y. (2015). Toward the rational design of non-precious transition metal oxides for oxygen electrocatalysis. Energy Environ. Sci..

[199] Lee D.U., Xu P., Cano Z.P., Kashkooli A.G., Park M.G., Chen Z. (2016). Recent progress and perspectives on bi-functional oxygen electrocatalysts for advanced rechargeable metal–air batteries. J. Mater. Chem. A.

[200] Hua B., Li M., Sun Y.-F., Zhang Y.-Q., Yan N., Chen J., Thundat T., Li J., Luo J.-L. (2017). A coupling for success: Controlled growth of Co/CoOx nanoshoots on perovskite mesoporous nanofibres as high-performance trifunctional electrocatalysts in alkaline condition. Nano Energy.

[201] Li S., Xia T., Dou Y., Xie Y., Wang J., Zhao H., Huo L. (2022). Phosphatizing Engineering of Perovskite Oxide Nanofibers for Hydrogen Evolution Reaction to Achieve Extraordinary Electrocatalytic Performance. Adv. Funct. Mater..

[202] Kim S.-j., Jung H., Lee C., Kim M.H., Lee Y. (2019). Comparative Study on Hydrogen Evolution Reaction Activity of Electrospun Nanofibers with Diverse Metallic Ir and IrO_2_ Composition Ratios. ACS Sustain. Chem. Eng..

[203] Fan L., Li Q., Wang D., Meng T., Yan M., Xing Z., Wang E., Yang X. (2020). Electrospun Ru–RuO_2_/MoO_3_ carbon nanorods with multi-active components: A Pt-like catalyst for the hydrogen evolution reaction. Chem. Commun..

[204] Wang J., Cui W., Liu Q., Xing Z., Asiri A.M., Sun X. (2016). Recent Progress in Cobalt-Based Heterogeneous Catalysts for Electrochemical Water Splitting. Adv. Mater..

[205] Zhu R., Chen F., Wang J., Song Y., Cheng J., Mao M., Ma H., Lu J., Cheng Y. (2020). Multi-channel V-doped CoP hollow nanofibers as high-performance hydrogen evolution reaction electrocatalysts. Nanoscale.

[206] Wei Y., Xu G., Wei Y., Ji L., Wang T., Liu Z., Wang S. (2022). Temperature-controlled synthesis of heterostructured Ru-Ru2P nanoparticles embedded in carbon nanofibers for highly efficient hydrogen production. Sci. China Mater..

[207] Kumaravel S., Karthick K., Sam Sankar S., Karmakar A., Madhu R., Bera K., Kundu S. (2021). Recent Progresses in Engineering of Ni and Co based Phosphides for Effective Electrocatalytic Water Splitting. ChemElectroChem.

[208] Tong J., Li Y., Bo L., Li W., Li T., Zhang Q., Kong D., Wang H., Li C. (2019). CoP/N-Doped Carbon Hollow Spheres Anchored on Electrospinning Core–Shell N-Doped Carbon Nanofibers as Efficient Electrocatalysts for Water Splitting. ACS Sustain. Chem. Eng..

[209] Hu J., Qin Y., Sun H., Ma Y., Lin L., Peng Y., Zhong J., Chen M., Zhao X., Deng Z. (2022). Combining Multivariate Electrospinning with Surface MOF Functionalization to Construct Tunable Active Sites toward Trifunctional Electrocatalysis. Small.

[210] Xie X.-Q., Liu J., Gu C., Li J., Zhao Y., Liu C.-S. (2022). Hierarchical structured CoP nanosheets/carbon nanofibers bifunctional eletrocatalyst for high-efficient overall water splitting. J. Energy Chem..

[211] Ren A., Yu B., Huang M., Liu Z. (2024). Encapsulation of cobalt prussian blue analogue-derived ultra-small CoP nanoparticles in electrospun N-doped porous carbon nanofibers as an efficient bifunctional electrocatalyst for water splitting. Int. J. Hydrogen Energy.

[212] Miao Y.-E., Li F., Zhou Y., Lai F., Lu H., Liu T. (2017). Engineering a nanotubular mesoporous cobalt phosphide electrocatalyst by the Kirkendall effect towards highly efficient hydrogen evolution reactions. Nanoscale.

[213] Yu M., Guo X., Chang X., Ma X., Zhang M. (2022). Assembled cobalt phosphide nanoparticles on carbon nanofibers as a bifunctional catalyst for hydrogen evolution reaction and oxygen evolution reaction. Sustain. Energy Fuels.

[214] Zhu X., Zhang W., Wang X., Ren A., Huang M., Ramakrishna S., Liu Z. (2023). Prussian blue analog derived CoP nanoparticles decorated on electrospun carbon nanofibers for efficient hydrogen evolution. J. Alloys Compd..

[215] Chen X., Yu B., Dong Y., Zhu X., Zhang W., Ramakrishna S., Liu Z. (2022). Electrospun porous carbon nanofibers decorated with iron-doped cobalt phosphide nanoparticles for hydrogen evolution. J. Alloys Compd..

[216] Li Y., Li H., Cao K., Jin T., Wang X., Sun H., Ning J., Wang Y., Jiao L. (2018). Electrospun three dimensional Co/CoP@nitrogen-doped carbon nanofibers network for efficient hydrogen evolution. Energy Storage Mater..

[217] Quan X., Ouyang C., Pan Y., Zhang C., Wu Z., Hong Z., Zhi M. (2020). Electrospinning metal Phosphide/Carbon nanofibers from Phytic Acid for hydrogen evolution reaction catalysts. Nanotechnology.

[218] Streckova M., Mudra E., Orinakova R., Markusova-Buckova L., Sebek M., Kovalcikova A., Sopcak T., Girman V., Dankova Z., Micusik M. (2016). Nickel and nickel phosphide nanoparticles embedded in electrospun carbon fibers as favourable electrocatalysts for hydrogen evolution. Chem. Eng. J..

[219] Streckova M., Orinakova R., Hovancova J., Kobera L., Brus J., Hungria A.B., Girman V., Mudra E., Heckova M., Podobova M. (2019). Fibrous electrocatalytic materials based on carbon/copper/copper phosphides for effective hydrogen evolution. Appl. Surf. Sci..

[220] Yang Y., Yang P., Zhou L., He R., Hao Y., Wang J., Qiu R., Zhao X., Yang L. (2021). Electrospun IrP2-carbon nanofibers for hydrogen evolution reaction in alkaline medium. Appl. Surf. Sci..

[221] Cao B., Cheng Y., Hu M., Jing P., Ma Z., Liu B., Gao R., Zhang J. (2019). Efficient and Durable 3D Self-Supported Nitrogen-Doped Carbon-Coupled Nickel/Cobalt Phosphide Electrodes: Stoichiometric Ratio Regulated Phase- and Morphology-Dependent Overall Water Splitting Performance. Adv. Funct. Mater..

[222] Zhang H., Li X., Hähnel A., Naumann V., Lin C., Azimi S., Schweizer S.L., Maijenburg A.W., Wehrspohn R.B. (2018). Bifunctional Heterostructure Assembly of NiFe LDH Nanosheets on NiCoP Nanowires for Highly Efficient and Stable Overall Water Splitting. Adv. Funct. Mater..

[223] Zhao Z., Yuan Z., Fang Z., Jian J., Li J., Yang M., Mo C., Zhang Y., Hu X., Li P. (2018). In Situ Activating Strategy to Significantly Boost Oxygen Electrocatalysis of Commercial Carbon Cloth for Flexible and Rechargeable Zn-Air Batteries. Adv. Sci..

[224] Streckova M., Petrus O., Guboova A., Orinakova R., Girman V., Bera C., Batkova M., Balaz M., Shepa J., Dusza J. (2022). Nanoarchitectonics of binary transition metal phosphides embedded in carbon fibers as a bifunctional electrocatalysts for electrolytic water splitting. J. Alloys Compd..

[225] Ďurovič M., Hnát J., Strečková M., Bouzek K. (2023). Efficient cathode for the hydrogen evolution reaction in alkaline membrane water electrolysis based on NiCoP embedded in carbon fibres. J. Power Sources.

[226] Wei B., Xu G., Hei J., Zhang L., Huang T. (2021). PBA derived FeCoP nanoparticles decorated on NCNFs as efficient electrocatalyst for water splitting. Int. J. Hydrogen Energy.

[227] Gong Y., Xu L.-H., Li J., Shan D. (2021). Confinement of transition metal phosphides in N, P-doped electrospun carbon fibers for enhanced electrocatalytic hydrogen evolution. J. Alloys Compd..

[228] Huang T., Xu G., Ding H., Zhang L., Wei B., Liu X. (2022). Ultrafine cobalt molybdenum phosphide nanoparticles embedded in crosslinked nitrogen-doped carbon nanofiber as efficient bifunctional catalyst for overall water splitting. J. Colloid Interface Sci..

[229] Yang L., Xu P., Zhang W., Chen M., Feng C., Yuan X. (2020). Facile synthesis of one-dimensional MoWP hybrid nanowires and their enhanced electrochemical catalytic activities. Chem. Phys. Lett..

[230] Surendran S., Shanmugapriya S., Sivanantham A., Shanmugam S., Kalai Selvan R. (2018). Electrospun Carbon Nanofibers Encapsulated with NiCoP: A Multifunctional Electrode for Supercapattery and Oxygen Reduction, Oxygen Evolution, and Hydrogen Evolution Reactions. Adv. Energy Mater..

[231] Mo Q., Zhang W., He L., Yu X., Gao Q. (2019). Bimetallic Ni2-xCoxP/N-doped carbon nanofibers: Solid-solution-alloy engineering toward efficient hydrogen evolution. Appl. Catal. B Environ..

[232] Sun X., Wei P., Zhang J., Gu S., Yang R., Fang C., Li Q., Han J., Jiang Z., He J. (2021). P, N-codoped carbon nanofibers confined ultra-small bimetallic NiCoP for highly efficient overall water splitting. Appl. Surf. Sci..

[233] Chen J., Huang F., Ke S., Shen J., Li Y., Zheng F., Li S. (2022). A dual-confinement strategy to construct cobalt-based phosphide nanoclusters within carbon nanofibers for bifunctional water splitting electrocatalysts. Dalton Trans..

[234] Wei B., Xu G., Hei J., Zhang L., Huang T., Wang Q. (2021). CoFeP hierarchical nanoarrays supported on nitrogen-doped carbon nanofiber as efficient electrocatalyst for water splitting. J. Colloid Interface Sci..

[235] Chen J., Zhang H., Yu J., Guan D., She S., Zhou W., Shao Z. (2021). Self-catalyzed formation of strongly interconnected multiphase molybdenum-based composites for efficient hydrogen evolution. Carbon Energy.

[236] Chen X., Le F., Lu Z., Zhou D., Yao H., Jia W. (2023). Ultrafine Electrospun Cobalt-Molybdenum Bimetallic Nitride as a Durable Electrocatalyst for Hydrogen Evolution. Inorg. Chem..

[237] Tian D., Denny S.R., Li K., Wang H., Kattel S., Chen J.G. (2021). Density functional theory studies of transition metal carbides and nitrides as electrocatalysts. Chem. Soc. Rev..

[238] Gao B., Li X., Ding K., Huang C., Li Q., Chu P.K., Huo K. (2019). Recent progress in nanostructured transition metal nitrides for advanced electrochemical energy storage. J. Mater. Chem. A.

[239] Xie J., Xie Y. (2016). Transition Metal Nitrides for Electrocatalytic Energy Conversion: Opportunities and Challenges. Chem. A Eur. J..

[240] Theerthagiri J., Lee S.J., Murthy A.P., Madhavan J., Choi M.Y. (2020). Fundamental aspects and recent advances in transition metal nitrides as electrocatalysts for hydrogen evolution reaction: A review. Curr. Opin. Solid State Mater. Sci..

[241] Peng X., Pi C., Zhang X., Li S., Huo K., Chu P.K. (2019). Recent progress of transition metal nitrides for efficient electrocatalytic water splitting. Sustain. Energy Fuels.

[242] Mukkavilli R.S., Ichangi A., Thiyagarajan G.B., Vollnhals F., Wilhelm M., Bhardwaj A., Christiansen S., Neelakantan L., Mathur S., Kumar R. (2022). Electrospun 1D Ta_3_N_5_ -(O) nanofibers as advanced electrocatalysts for hydrogen evolution reaction in proton exchange membrane water electrolyser. Open Ceram..

[243] Wan C., Regmi Y.N., Leonard B.M. (2014). Multiple Phases of Molybdenum Carbide as Electrocatalysts for the Hydrogen Evolution Reaction. Angew. Chem. Int. Ed..

[244] Gao Q., Zhang W., Shi Z., Yang L., Tang Y. (2019). Structural Design and Electronic Modulation of Transition-Metal-Carbide Electrocatalysts toward Efficient Hydrogen Evolution. Adv. Mater..

[245] Lu X., Li M., Wang H., Wang C. (2019). Advanced electrospun nanomaterials for highly efficient electrocatalysis. Inorg. Chem. Front..

[246] Gao W., Shi Y., Zuo L., Fan W., Liu T. (2016). Rough-surfaced molybdenum carbide nanobeads grown on graphene-coated carbon nanofibers membrane as free-standing hydrogen evolution reaction electrocatalyst. Mater. Today Chem..

[247] Li M., Wang H., Zhu Y., Tian D., Wang C., Lu X. (2019). Mo/Mo_2_C encapsulated in nitrogen-doped carbon nanofibers as efficiently integrated heterojunction electrocatalysts for hydrogen evolution reaction in wide pH range. Appl. Surf. Sci..

[248] Zhang Y., Kong D., Bo L., Shi W., Guan X., Wang Y., Lei Z., Tong J. (2021). Electrospinning Preparation of N, P Dual-Doped Molybdenum Carbide/Porous Carbon Fibers with Highly Improved Electrocatalytic Activity for Hydrogen Evolution Reaction. ACS Appl. Energy Mater..

[249] Yuan X., Huang W., Kong L., Guo S., Cheng Y. (2021). Ditungsten carbide nanoparticles homogeneously embedded in carbon nanofibers for efficient hydrogen production. Chem. Eng. J..

[250] Ji D., Peng S., Lu J., Li L., Yang S., Yang G., Qin X., Srinivasan M., Ramakrishna S. (2017). Design and synthesis of porous channel-rich carbon nanofibers for self-standing oxygen reduction reaction and hydrogen evolution reaction bifunctional catalysts in alkaline medium. J. Mater. Chem. A.

[251] Ouyang T., Chen A.-N., He Z.-Z., Liu Z.-Q., Tong Y. (2018). Rational design of atomically dispersed nickel active sites in β-Mo_2_C for the hydrogen evolution reaction at all pH values. Chem. Commun..

[252] Han W., Chen L., Ma B., Wang J., Song W., Fan X., Li Y., Zhang F., Peng W. (2019). Ultra-small Mo_2_C nanodots encapsulated in nitrogen-doped porous carbon for pH-universal hydrogen evolution: Insights into the synergistic enhancement of HER activity by nitrogen doping and structural defects. J. Mater. Chem. A.

[253] Xiao P., Ge X., Wang H., Liu Z., Fisher A., Wang X. (2015). Novel Molybdenum Carbide–Tungsten Carbide Composite Nanowires and Their Electrochemical Activation for Efficient and Stable Hydrogen Evolution. Adv. Funct. Mater..

[254] Wang J., Zhu R., Cheng J., Song Y., Mao M., Chen F., Cheng Y. (2020). Co, Mo2C encapsulated in N-doped carbon nanofiber as self-supported electrocatalyst for hydrogen evolution reaction. Chem. Eng. J..

[255] Zhang L., Wei K., Ma J., Wang J., Liu Z., Xing R., Jiao T. (2021). Coupled Sn/Mo2C nanoparticles wrapped in carbon nanofibers by electrospinning as high-performance electrocatalyst for hydrogen evolution reaction. Appl. Surf. Sci..

[256] Sun J., Liu J., Chen H., Han X., Wu Y., He J., Han C., Yang G., Shan Y. (2020). Strongly coupled Mo_2_C and Ni nanoparticles with in-situ formed interfaces encapsulated by porous carbon nanofibers for efficient hydrogen evolution reaction under alkaline conditions. J. Colloid Interface Sci..

[257] Yang P., Zhao H., Yang Y., Zhao P., Zhao X., Yang L. (2020). Fabrication of N, P-codoped Mo_2_C/Carbon Nanofibers via Electrospinning as Electrocatalyst for Hydrogen&nbsp;Evolution Reaction. ES Mater. Manuf..

[258] Pan X., Lu S., Zhang D., Zhang Y., Duan F., Zhu H., Gu H., Wang S., Du M. (2020). Atom-precise incorporation of platinum into ultrafine transition metal carbides for efficient synergetic electrochemical hydrogen evolution. J. Mater. Chem. A.

[259] Li M., Zhu Y., Wang H., Wang C., Pinna N., Lu X. (2019). Ni Strongly Coupled with Mo_2_C Encapsulated in Nitrogen-Doped Carbon Nanofibers as Robust Bifunctional Catalyst for Overall Water Splitting. Adv. Energy Mater..

[260] Heckova M., Streckova M., Orinakova R., Hovancova J., Guboova A., Sopcak T., Kovalcikova A., Plesingerova B., Medved D., Szabo J. (2020). Porous carbon fibers for effective hydrogen evolution. Appl. Surf. Sci..

[261] Gao Y., Xiao Z., Kong D., Iqbal R., Yang Q.-H., Zhi L. (2019). N,P co-doped hollow carbon nanofiber membranes with superior mass transfer property for trifunctional metal-free electrocatalysis. Nano Energy.

[262] Sun J., Ge Q., Guo L., Yang Z. (2020). Nitrogen doped carbon fibers derived from carbonization of electrospun polyacrylonitrile as efficient metal-free HER electrocatalyst. Int. J. Hydrogen Energy.

[263] Li H., Ren B., Liu W., Jing L., Tay R.Y., Tsang S.H., Ricardez–Sandoval L., Yu A., Teo E.H.T. (2021). Boron nanosheets induced microstructure and charge transfer tailoring in carbon nanofibrous mats towards highly efficient water splitting. Nano Energy.

[264] Li J., Qian J., Chen X., Zeng X., Li L., Ouyang B., Kan E., Zhang W. (2022). Three-dimensional hierarchical graphitic carbon encapsulated CoNi alloy/N-doped CNTs/carbon nanofibers as an efficient multifunctional electrocatalyst for high-performance microbial fuel cells. Compos. Part B Eng..

[265] Zhang Y., Shi W., Bo L., Shen Y., Ji X., Xia L., Guan X., Wang Y., Tong J. (2022). Electrospinning construction of heterostructural Co_3_W_3_C/CoP nanoparticles embedded in N, P-doped hierarchically porous carbon fibers as excellent multifunctional electrocatalyst for Zn-air batteries and water splitting. Chem. Eng. J..

[266] Gu L., Zhu H., Yu D., Zhang S., Chen J., Wang J., Wan M., Zhang M., Du M. (2017). A Facile Strategy to Synthesize Cobalt-Based Self-Supported Material for Electrocatalytic Water Splitting. Part. Part. Syst. Charact..

[267] Sun C., Wang C., Xie H., Han G., Zhang Y., Zhao H. (2023). 2D Cobalt Chalcogenide Heteronanostructures Enable Efficient Alkaline Hydrogen Evolution Reaction. Small.

[268] Mugheri A.Q., Ali S., Narejo G.S., Otho A.A., Lal R., Abro M.A., Memon S.H., Abbasi F. (2020). Electrospun fibrous active bimetallic electrocatalyst for hydrogen evolution. Int. J. Hydrogen Energy.

[269] El-Maghrabi H.H., Nada A.A., Bekheet M.F., Roualdes S., Riedel W., Iatsunskyi I., Coy E., Gurlo A., Bechelany M. (2021). Coaxial nanofibers of nickel/gadolinium oxide/nickel oxide as highly effective electrocatalysts for hydrogen evolution reaction. J. Colloid Interface Sci..

[270] Liu Z., Guo F., Han L., Xiao J., Zeng X., Zhang C., Dong P., Li M., Zhang Y. (2022). Manganese Oxide/Iron Carbide Encapsulated in Nitrogen and Boron Codoped Carbon Nanowire Networks as Accelerated Alkaline Hydrogen Evolution and Oxygen Reduction Bifunctional Electrocatalysts. ACS Appl. Mater. Interfaces.

[271] Nagappan S., Karmakar A., Madhu R., N Dhandapani H., Bera K., De A., Kundu S. (2022). Electronically Modified Ce3+ Ion Doped 2D NiFe-LDH Nanosheets over a 1D Microfiber: A High-Performance Electrocatalyst for Overall Water Splitting. ACS Appl. Energy Mater..

[272] Chen X., Li W., Wang C., Lu X. (2023). Wet chemical synthesis of rhodium nanoparticles anchored on cobalt/nitrogen-doped carbon nanofibers for high-performance alkaline and acidic hydrogen evolution. J. Colloid Interface Sci..

[273] Zhang C.-L., Xie Y., Liu J.-T., Cao F.-H., Cong H.-P., Li H. (2021). 1D Core−Shell MOFs derived CoP Nanoparticles-Embedded N-doped porous carbon nanotubes anchored with MoS2 nanosheets as efficient bifunctional electrocatalysts. Chem. Eng. J..

[274] Liu H., Sun J., Xu Z., Zhou W., Han C., Yang G., Shan Y. (2022). Ru nanoparticles decorated Ni-V_2_NO heterostructures in carbon nanofibers as efficient electrocatalysts for hydrogen evolution reaction. J. Electroanal. Chem..

[275] Zhang L., Yin J., Wei K., Li B., Jiao T., Chen Y., Zhou J., Peng Q. (2020). Fabrication of hierarchical SrTiO_3_@MoS_2_ heterostructure nanofibers as efficient and low-cost electrocatalysts for hydrogen-evolution reactions. Nanotechnology.

[276] Wan H., Liu X., Wang H., Ma R., Sasaki T. (2019). Recent advances in developing high-performance nanostructured electrocatalysts based on 3d transition metal elements. Nanoscale Horiz..

[277] Luo X., Zhou Q., Du S., Li J., Zhong J., Deng X., Liu Y. (2018). Porous Co_9_S_8_/Nitrogen, Sulfur-Doped Carbon@Mo_2_C Dual Catalyst for Efficient Water Splitting. ACS Appl. Mater. Interfaces.

[278] Zhang T., Yu J., Guo H., Liu J., Liu Q., Song D., Chen R., Li R., Liu P., Wang J. (2020). Heterogeneous CoSe_2_–CoO nanoparticles immobilized into N-doped carbon fibers for efficient overall water splitting. Electrochim. Acta.

[279] Long X., Lin H., Zhou D., An Y., Yang S. (2018). Enhancing Full Water-Splitting Performance of Transition Metal Bifunctional Electrocatalysts in Alkaline Solutions by Tailoring CeO2–Transition Metal Oxides–Ni Nanointerfaces. ACS Energy Lett..

[280] Wang C., Lu H., Mao Z., Yan C., Shen G., Wang X. (2020). Bimetal Schottky Heterojunction Boosting Energy-Saving Hydrogen Production from Alkaline Water via Urea Electrocatalysis. Adv. Funct. Mater..

[281] Chen S., Wang C., Liu S., Huang M., Lu J., Xu P., Tong H., Hu L., Chen Q. (2021). Boosting Hydrazine Oxidation Reaction on CoP/Co Mott–Schottky Electrocatalyst through Engineering Active Sites. J. Phys. Chem. Lett..

[282] Wang J., Liao T., Wei Z., Sun J., Guo J., Sun Z. (2021). Heteroatom-Doping of Non-Noble Metal-Based Catalysts for Electrocatalytic Hydrogen Evolution: An Electronic Structure Tuning Strategy. Small Methods.

[283] Wang M., Zhang C., Meng T., Pu Z., Jin H., He D., Zhang J., Mu S. (2019). Iron oxide and phosphide encapsulated within N,P-doped microporous carbon nanofibers as advanced tri-functional electrocatalyst toward oxygen reduction/evolution and hydrogen evolution reactions and zinc-air batteries. J. Power Sources.

[284] Song Y., Cheng J., Liu J., Ye Q., Gao X., Lu J., Cheng Y. (2021). Modulating electronic structure of cobalt phosphide porous nanofiber by ruthenium and nickel dual doping for highly-efficiency overall water splitting at high current density. Appl. Catal. B Environ..

[285] Guo Y., Zhang X., Zhang X., You T. (2015). Defect- and S-rich ultrathin MoS_2_ nanosheet embedded N-doped carbon nanofibers for efficient hydrogen evolution. J. Mater. Chem. A.

[286] Zhu Y., Song L., Song N., Li M., Wang C., Lu X. (2019). Bifunctional and Efficient CoS_2_–C@MoS_2_ Core–Shell Nanofiber Electrocatalyst for Water Splitting. ACS Sustain. Chem. Eng..

[287] Zhang S., Li Y., Zhu H., Lu S., Ma P., Dong W., Duan F., Chen M., Du M. (2020). Understanding the Role of Nanoscale Heterointerfaces in Core/Shell Structures for Water Splitting: Covalent Bonding Interaction Boosts the Activity of Binary Transition-Metal Sulfides. ACS Appl. Mater. Interfaces.

[288] Chen W., Lan W., Wang H., Zhang A., Liu C. (2022). Engineering of sugarcane bagasse based porous carbon nanofiber-supported the CoP/Co2P heterostructure for efficient overall water splitting. Electrochim. Acta.

[289] Zhang Y.-Q., Tao H.-B., Chen Z., Li M., Sun Y.-F., Hua B., Luo J.-L. (2019). In situ grown cobalt phosphide (CoP) on perovskite nanofibers as an optimized trifunctional electrocatalyst for Zn–air batteries and overall water splitting. J. Mater. Chem. A.

[290] Li T., Yin J., Sun D., Zhang M., Pang H., Xu L., Zhang Y., Yang J., Tang Y., Xue J. (2022). Manipulation of Mott−Schottky Ni/CeO_2_ Heterojunctions into N-Doped Carbon Nanofibers for High-Efficiency Electrochemical Water Splitting. Small.

[291] Lin H., Zhang W., Shi Z., Che M., Yu X., Tang Y., Gao Q. (2017). Electrospinning Hetero-Nanofibers of Fe_3_C-Mo_2_C/Nitrogen-Doped-Carbon as Efficient Electrocatalysts for Hydrogen Evolution. ChemSusChem.

[292] Gong T., Zhang J., Liu Y., Hou L., Deng J., Yuan C. (2023). Construction of hetero-phase Mo_2_C-CoO@N-CNFs film as a self-supported Bi-functional catalyst towards overall water splitting. Chem. Eng. J..

[293] Li F., Xu R., Li Y., Liang F., Zhang D., Fu W.-F., Lv X.-J. (2019). N-doped carbon coated NiCo_2_S_4_ hollow nanotube as bifunctional electrocatalyst for overall water splitting. Carbon.

[294] Zheng Y., Hu H., Zhu Y., Rong J., Zhang T., Yang D., Wen Q., Qiu F. (2022). ZIF-67-Derived (NiCo)S_2_@NC Nanosheet Arrays Hybrid for Efficient Overall Water Splitting. Inorg. Chem..

[295] Guo D., Wang J., Zhang L., Chen X., Wan Z., Xi B. (2020). Strategic Atomic Layer Deposition and Electrospinning of Cobalt Sulfide/Nitride Composite as Efficient Bifunctional Electrocatalysts for Overall Water Splitting. Small.

[296] Zhang S., Gao G., Hao J., Wang M., Zhu H., Lu S., Duan F., Dong W., Du M., Zhao Y. (2019). Low-Electronegativity Vanadium Substitution in Cobalt Carbide Induced Enhanced Electron Transfer for Efficient Overall Water Splitting. ACS Appl. Mater. Interfaces.

[297] Zhang H., Wang L., He W., Liu D., Shao H., Yu W., Yin D., Dong X. (2022). Mo_2_C regulated by cobalt components doping in N-doped hollow carbon nanofibers as an efficient electrocatalyst for hydrogen evolution reaction. Int. J. Hydrogen Energy.

[298] Ji C., Yang G., Ilango P.R., Song J., Yu D., Han S., Zhang D., Li L., Peng S. (2020). Molybdenum Carbide-Embedded Multichannel Hollow Carbon Nanofibers as Bifunctional Catalysts for Water Splitting. Chem. Asian J..

